# Research hotspots and frontiers of application of mass spectrometry breath test in respiratory diseases

**DOI:** 10.3389/fmed.2025.1618588

**Published:** 2025-08-13

**Authors:** Yunanji Zhou, Xinyi Qiu, Ting Yuan, Qian Wang, Lei Du, Lihua Wang, Zhaohui Ding

**Affiliations:** ^1^Qi Huang Chinese Medicine Academy, Jiangxi University of Chinese Medicine, Nanchang, China; ^2^School of Clinical Medicine, Jiangxi University of Chinese Medicine, Nanchang, China; ^3^Department of Pulmonary Disease, Affiliated Hospital of Jiangxi University of Traditional Chinese Medicine, Nanchang, China

**Keywords:** mass spectrometry, breath test, respiratory diseases, volatile organic compounds, bibliometrics, biomarkers

## Abstract

Mass spectrometry (MS)-based breath analysis has emerged as a promising non-invasive approach for diagnosing and monitoring respiratory diseases through the identification of volatile organic compounds (VOCs). This study conducted a comprehensive bibliometric analysis of 467 publications (2003–2024) to map global research trends, influential contributors, and thematic hotspots in this field. Results showed a sustained annual growth rate of 11.03%, with the United States, the United Kingdom, the Netherlands, and China leading in publication output and institutional collaborations. Key research areas included VOC profiling for COPD, asthma, lung cancer, and COVID-19, as well as advances in real-time MS techniques and machine learning-based data interpretation. Co-citation analysis revealed a shift toward precision medicine and multi-omics integration, underscoring the field’s transition from discovery to clinical translation. Despite challenges in standardization and reproducibility, MS-based breathomics holds transformative potential for respiratory diagnostics. This study provides a roadmap for future research priorities, emphasizing the need for interdisciplinary collaboration, composite biomarker validation, and artificial intelligence integration.

## 1 Introduction

By detecting the mass-to-charge ratio (m/z) values of ions and combining them with fragment ion spectra, mass spectrometry (MS) can qualitatively provide information on the molecular weight and structural features of substances. Target substances can also be quantitatively analyzed using MS by measuring the abundance of particular m/z ions in conjunction with internal standard procedures or standard curves (1). Because MS supports a range of ionization techniques (such as electrospray ionization and electron bombardment ionization) and can be combined with separation techniques like gas chromatography (GC) and liquid chromatography (LC), it has great potential and value in clinical practice. This makes it feasible for the analysis of complex samples for use in clinical biological analysis (2). Respiratory disorders are closely linked to volatile organic compounds (VOCs) in exhaled breath. The application of MS to identify VOCs has grown in popularity in recent years.

**Figure 1:**
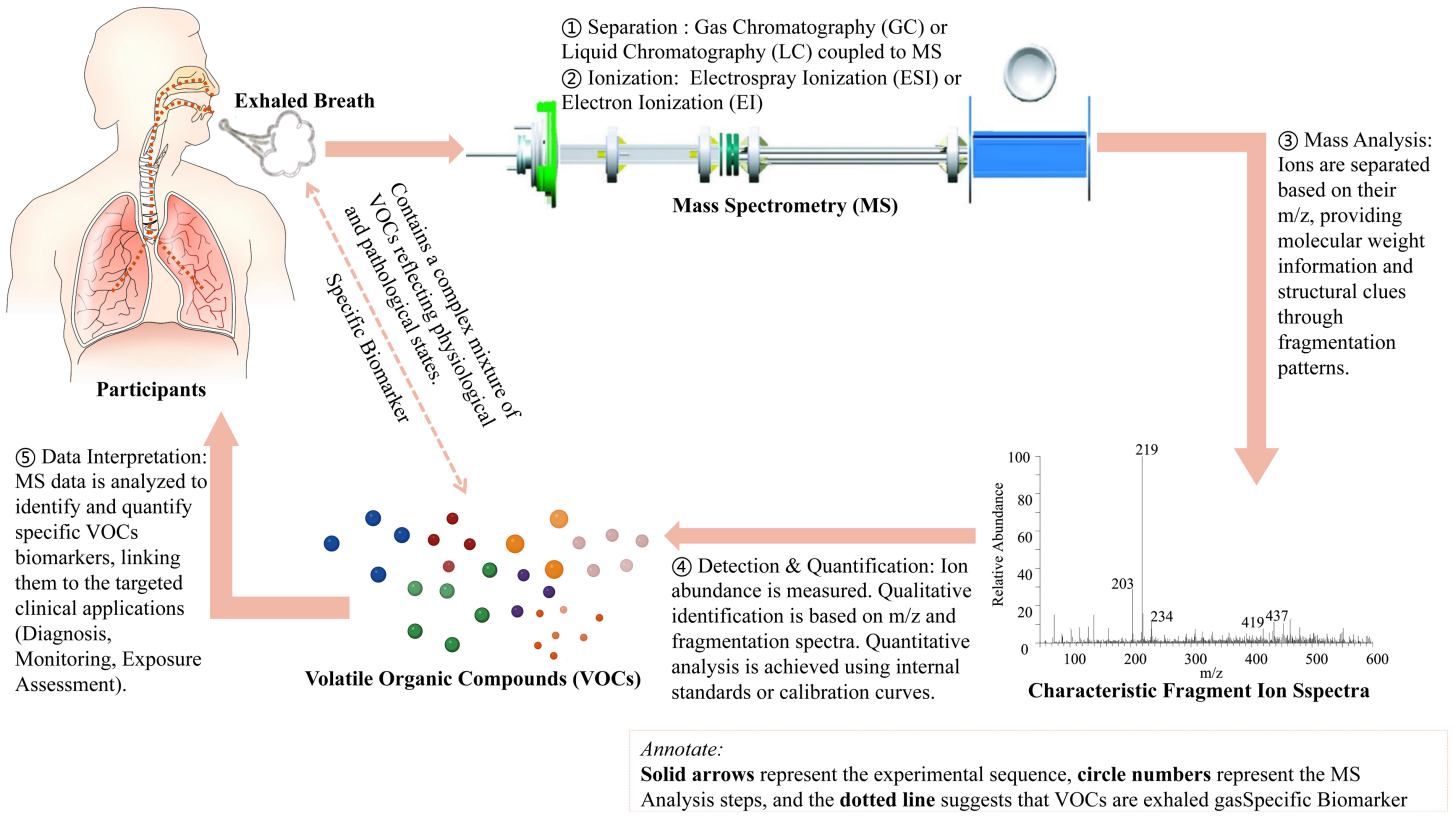
Schematic of exhaled gas mass spectrometry for the detection of VOCs.

Figure 1 schematically outlines the integrated workflow for clinical biomarker discovery and analysis using MS-based profiling of VOCs in exhaled breath. The process initiates with participant sampling, where exhaled breath is collected as a complex matrix rich in VOCs originating from systemic metabolism. The MS analysis phase involves four critical steps: (1) Sample Introduction & Separation (optional but common), typically employing online-coupled GC/LC–MS to resolve complex VOC mixtures (2) (2) Ionization using techniques such as electrospray ionization (ESI, for LC–MS) or electron ionization (EI, for GC–MS) to generate gas-phase ions (2) (3) Mass Analysis, where ions are separated by m/z to determine molecular weights, with tandem MS fragment ion spectra providing structural elucidation (1); and (4) Detection and Quantification, enabling qualitative identification (via m/z and fragmentation patterns) and precise quantitation (using internal standards or calibration curves) (1). Finally, computational interpretation of MS data (spectra/chromatograms) correlates quantified VOC biomarkers with clinical endpoints, facilitating applications in disease diagnosis, metabolic and drug monitoring, and environmental exposure assessment (3–5).

Coupled MS offers distinct advantages over traditional detection techniques, including non-invasiveness, high sensitivity, high specificity, and real-time monitoring capabilities. While biopsies provide histological confirmation, they carry risks of pneumothorax (15–25% incidence) and are unsuitable for serial monitoring. Conversely, MS breath analysis enables real-time, repeatable assessment of metabolic activity but lacks spatial resolution for tumor localization. Conventional imaging techniques (e.g., CT/MRI) primarily provide anatomical information but lack sensitivity to functional metabolic changes during early disease stages. In contrast, MS detects molecular-level alterations by identifying metabolite structures, enabling lesion detection months before anatomical abnormalities manifest, thereby significantly improving early diagnosis rates. For instance, Wang et al. demonstrated that proton transfer reaction time-of-flight MS (PTR-TOF-MS) outperformed CT in diagnosing early-stage lung cancer, with respiratory analysis showing superior diagnostic performance (88.6% sensitivity, 63.6% specificity, 79.2% accuracy) versus CT (74.3, 59.1, 68.1%) (6). The integration of mass spectrometry with low-dose CT may enhance early detection by identifying metabolic changes that precede anatomical abnormalities. Breath analysis via MS is now extensively employed in respiratory medicine. MS-based multidimensional analysis facilitates not only disease diagnosis and monitoring, but also elucidates underlying physiological and pathological mechanisms (7). Bos et al. identified three exhaled VOCs—octane, acetaldehyde, and 3-methylheptane—using GC–MS as diagnostic biomarkers for acute respiratory distress syndrome (ARDS). They further hypothesized these compounds originate from cellular metabolism and lipid peroxidation (8). Gaugg et al. (9) employed high-resolution MS for real-time breath analysis to monitor metabolic changes following bronchodilator inhalation in asthma and chronic obstructive pulmonary disease (COPD) patients.

Bibliometrics employs statistical techniques to quantitatively analyze published literature (10). Using specialized analytical tools (e.g., CiteSpace, VOSviewer), this method extracts multivariate data—including authors, institutions, keywords, and citations—to visualize knowledge evolution and structural relationships. This quantitative approach provides an invaluable framework for examining research dynamics, identifying emerging trends, detecting knowledge gaps, and forecasting domain-specific research hotspots. We conducted a bibliometric analysis of breath test mass spectrometry literature (2003–2024) indexed in Web of Science (WoS) and PubMed. Through systematic data analysis, we: (1) identified seminal publications and key applications, (2) analyzed high-impact citations and keyword co-occurrences, (3) generated thematic clusters, (4) mapped current research hotspots, and (5) identified emerging frontiers—collectively elucidating the field’s developmental trajectory. This longitudinal analysis provides researchers with a comprehensive knowledge map of the global MS breath testing landscape for respiratory disorders, while establishing critical references for future investigations.

## 2 Materials and methods

We systematically searched WoS, PubMed, Scopus, and Embase for publications (2000–2024) on MS-based breath analysis in respiratory diseases. Preliminary screening established 2003 as the valid inception year, as empirical studies first appeared then. Pre-2003 publications contained only speculative perspectives lacking experimental validation.

Using R Studio, we exported records and removed duplicates across WOS, Scopus, and Embase via Excel’s COUNTIF algorithm. Duplication rates were: WOS-Scopus 92.1% (430/467) and WOS-Embase 89.1% (416/467). We standardized Scopus affiliations (recorded as secondary entities) via custom VBA scripting for hierarchical VOSviewer analysis. Scopus and Embase unique records were converted to WOS format using CiteSpace and merged. Integration validity was confirmed with negligible analytical impact.

Non-English publications (*n* = 14; 12 articles, 2 reviews) underwent full-text review. Eight contained novel content (7 articles, 1 review), while six duplicated English publications. These unique non-English records were excluded from network analyses due to metadata inconsistencies. Their minimal impact (<1.7% corpus coverage) was documented despite potential content scope expansion.

### 2.1 Data source and search strategy

Web of Science Core Collection (WoSCC) is widely recognized as a premier bibliographic database for bibliometric analysis, with established scholarly credibility (11). We systematically searched WoSCC and PubMed (January 1, 2003 - December 31, 2024) to retrieve relevant publications. The search strategy employed: TS = (“Mass spectrometry”) AND TS = (“Breath test”). Retrieval fields included title, abstract, author keywords, and references.

### 2.2 Manual screening process

#### 2.2.1 Screening and analysis workflow

Literature processing followed a tiered protocol: (1) A primary investigator retrieved and downloaded all articles; (2) Two independent researchers classified publications by study type and extracted metadata; (3) Two other researchers performed full-text critical appraisal and content synthesis; (4) Post-deduplication, five researchers conducted relevance screening to exclude irrelevant publications; (5) The primary investigator executed bibliometric analysis to ensure procedural consistency; (6) Discrepancies at each stage were resolved via consensus meetings with ≥80% agreement threshold.

#### 2.2.2 Inclusion criteria

(1) Literature containing breath tests and mass spectrometry; (2) Application scope of respiratory diseases; (3) Literature involving *in vitro* and *in vivo* experimental studies, clinical trial studies, reviews, and public database analysis studies; (4) Literature published in English; (5) Literature involving disease diagnosis, disease progression monitoring, drug treatment monitoring, and toxicology studies; (6) Books with full bibliographic details (title, author, country, keywords, and source).

#### 2.2.3 Exclusion criteria

(1) Duplicate publications; (2) Newspapers, patents, patents, conference papers, scientific and health literature, etc.; (3) Insufficient access to literature.

#### 2.2.4 Data standardization

Following screening, the papers were exported in Refworks and Plain Text File formats. Special symbols were eliminated. Keyword names were standardized; for example, “airway resistance” was merged with “airways.” Country/region names have been standardized; for example, “Northern Ireland,” “Wales,” “England,” and “Scotland” have been designated as “England” respectively. Institutional names were standardized; for example, “Peking univ. peoples hosp” was combined with “Peking univ.” The retrieved papers were then put together with the CiteSpace software’s Data Import/Export function.

### 2.3 Bibliometric analysis and visualization

**Figure 2:**
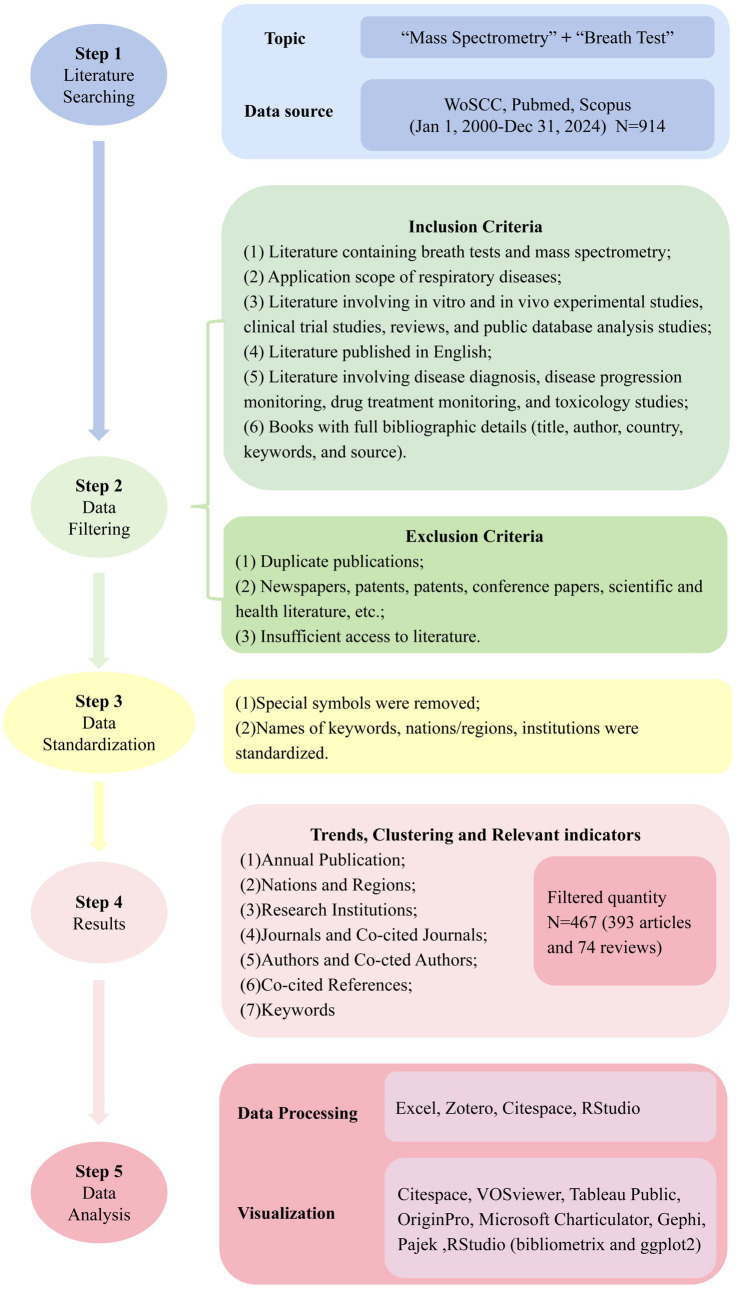
Workflow diagram of the literature review and analysis process.

From an initial retrieval of 914 documents, 467 publications (393 articles, 74 reviews) met inclusion criteria after deduplication (Zotero) and relevance screening. Analytical tool selection was based on specialized capabilities: CiteSpace (v6.2. R4) was employed for temporal evolution analysis (2003–2024) and burst detection due to its optimized algorithms for identifying emerging trends through time-slicing (1-year intervals) and pruning parameters (top 10%, Pathfinder network) (12, 13). VOSviewer (v1.6.19) generated co-occurrence/cluster networks (authors, institutions, journals) leveraging its superior clustering accuracy and visualization of large datasets. R Bibliometrix (v4.1.3) complemented quantitative analyses (H-index, citation metrics) through robust statistical modeling (ggplot2, reshape2 packages). Gephi/Pajek integration resolved complex network topologies (>500 nodes) where VOSviewer’s layout algorithms reached computational limits. Tableau Public enhanced geospatial mapping precision for country-level collaborations (14). OriginPro (v2023) produced publication trend fittings (polynomial curves) and heatmaps via its advanced numerical engine. This multi-tool approach ensured comprehensive coverage of bibliometric dimensions (temporal, structural, spatial), algorithmic cross-validation through complementary analytical methods, optimization of visualization clarity at varying data scales (12). The flowchart of our study is shown in Figure 2.

### 2.4 Research ethics

In this review, a bibliometric analysis was conducted. All data sources were available online and did not involve animal or human subjects. Therefore, no permission from the ethics committee was required.

## 3 Results

Table 1 quantitatively identifies leading entities across analytical dimensions—including country, organization, journals, authors and reference —within the MS-based breath analysis domain for respiratory pathologies.

**Table 1:** Bibliometrics top 3 of each section details.

Details		Top 3
Country		USA
		UK
		Netherlands
Organization		Univ Amsterdam
		Maastricht Univ
		Chinese Acad Sci
Journals	Highly published journals	Journal of Breath Research
		Scientific Reports
		European Respiratory Journal
	Highly cited journals	J Breath Res
		Am J Resp Crit Care
		Chest
	Co-cited journals	J Breath Res
		Eur Respir J
		Am J Resp Crit Care
Authors	Publishing author	Fowler, Stephen J.
		Schultz, Marcus J.
		Bos, Lieuwe D. J.
	Co-citations authors	Phillips, M
		Smith, D
		Montuschi, P
Reference	Citation document	Buszewski (124)
		Machado et al. (125)
		Phillips et al. (126)
	Co-citation cited reference	Phillips et al. (21)
		Pauling et al. (133)
		Miekisch et al. (20)

### 3.1 Trends of annual publication

**Figure 3:**
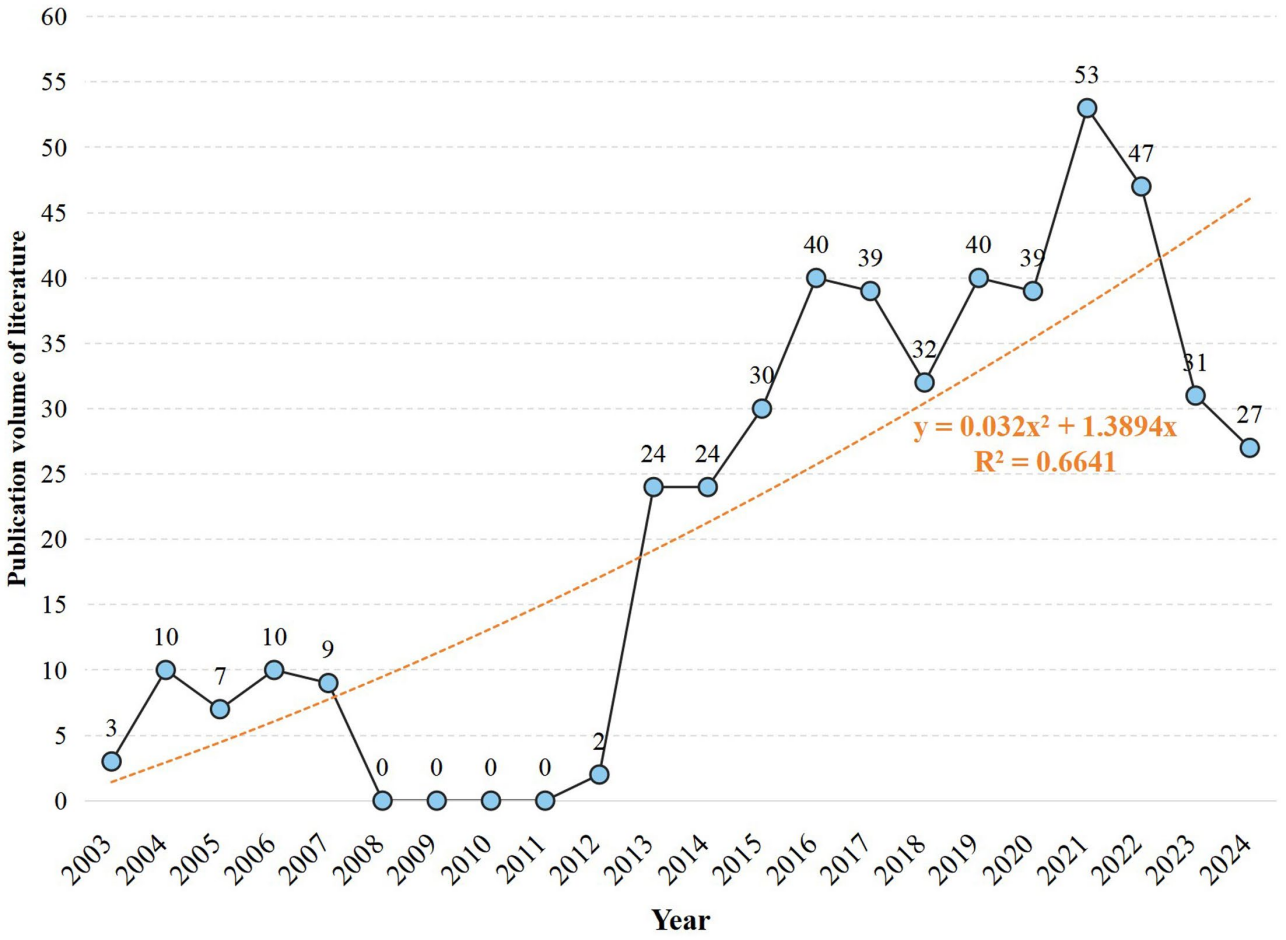
Publication growth over time: annual count and trend line (2003–2024).

Our bibliometric analysis identified 467 relevant publications in the MS-based breath analysis domain (2003–2024). The field emerged from Corradi et al.’s (15) landmark study quantifying aldehydes in COPD exhaled breath condensate. Publication output grew at 11.03% annually (Figure 3), evolving through four technological epochs:

Exploratory Phase (2003–2007; *n* = 39): Established foundational protocols building upon aldehyde detection in respiratory matrices (15). Constraint Phase (2008–2012; *n* = 41): Shifted to liquid-phase MS driven by gas-phase limitations: <50% chromatographic resolution for >15-component VOC mixtures and 20–40% analyte loss during storage (e.g., benzene adsorption in Tedlar® bags) (16). Breakthrough Phase (2013–2022; n = 426): Resolved prior constraints through: (i) SESI and PTR-MS enabling real-time detection without pre-concentration (17); (ii) ATS/ERS-standardized sampling reducing biological variance by >30% (18); (iii) Multicenter validations establishing diagnostic markers (e.g., acetone/ammonia ratio with 86% asthma sensitivity) (19). Maturation Phase (2023–2024; >27/yr): Focus transitioned to biomarker verification in multi-center cohorts and longitudinal validation, essential for clinical translation. Binomial growth modeling (R^2^ = 0.6641) predicts sustained expansion, contingent on overcoming point-of-care miniaturization and regulatory challenges.

### 3.2 Analysis by nations and regions

**Figure 4:**
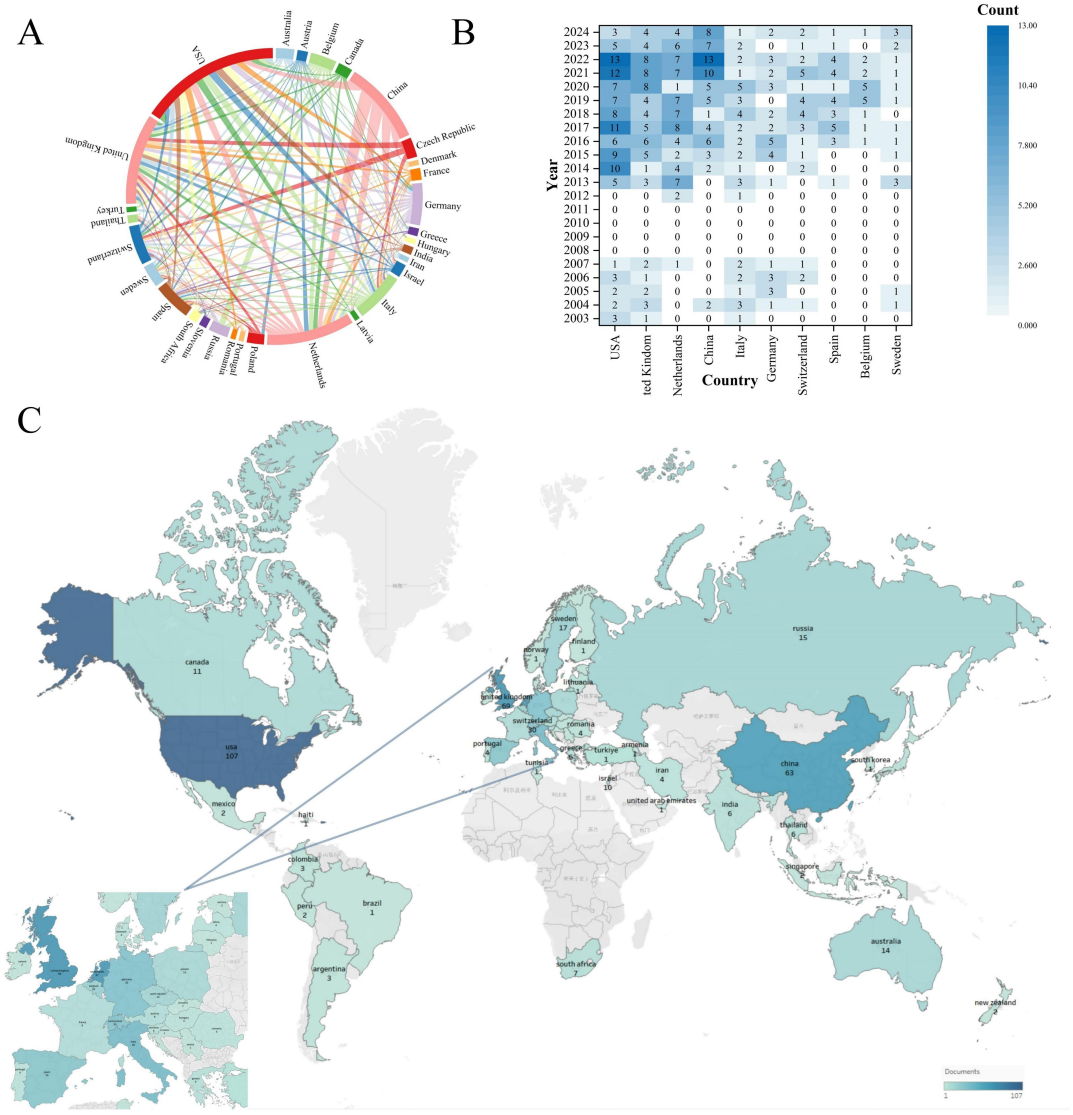
Global collaboration and research output in mass spectrometry breath analysis by country/region. **(A)** Network map of international collaborations: Node size = Number of publications from that country/region. Line thickness = Strength of collaboration between countries. **(B)** Leading countries/regions by publication volume over time: shows the annual output trends for the top 10 most productive countries/regions. **(C)** Global application focus: map highlighting where breath analysis for respiratory diseases is most actively researched.

Research participation spanned 54 countries/regions (2003–2024). Figure 4A maps international collaboration networks among countries with ≥3 publications. The UK maintained the highest collaboration count (n = 94), ahead of the Netherlands (*n* = 71) and USA (*n* = 59). Figure 4B tracks annual publication trends for the top 10 productive countries. Initial publications emerged from the USA and UK in 2003. Sustained output growth in the USA, UK, Netherlands, and China reflects established research capacity. Figure 4C visualizes global publication density, with intensity gradients indicating high-output regions. Table 2 quantifies research impact for the top 10 countries through publication volume, citation metrics, and collaboration indices. The USA led in absolute output (*n* = 107) and total citations (4,603), with 43.02 mean citations per paper. Italy achieved the highest mean citation rate (58.16 per article), suggesting disproportionate scientific influence. Global research distribution confirms MS breath analysis as an internationally significant respiratory diagnostic approach.

**Table 2:** Top 10 countries/regions by research output and influence.

Rank	Country	Documents	Citations	Cooperation intensity	Average citation per paper
1	USA	107	4,603	59	43.02
2	UK	69	2,817	94	40.83
3	Netherlands	67	2,917	71	43.54
4	China	63	1890	14	30.00
5	Italy	38	2,210	35	58.16
6	Germany	32	1,198	29	37.44
7	Switzerland	30	1,342	27	44.73
8	Spain	29	677	45	23.34
9	Belgium	20	701	25	35.05
10	Sweden	17	847	39	49.82

### 3.3 Analysis by research institutions

**Figure 5:**
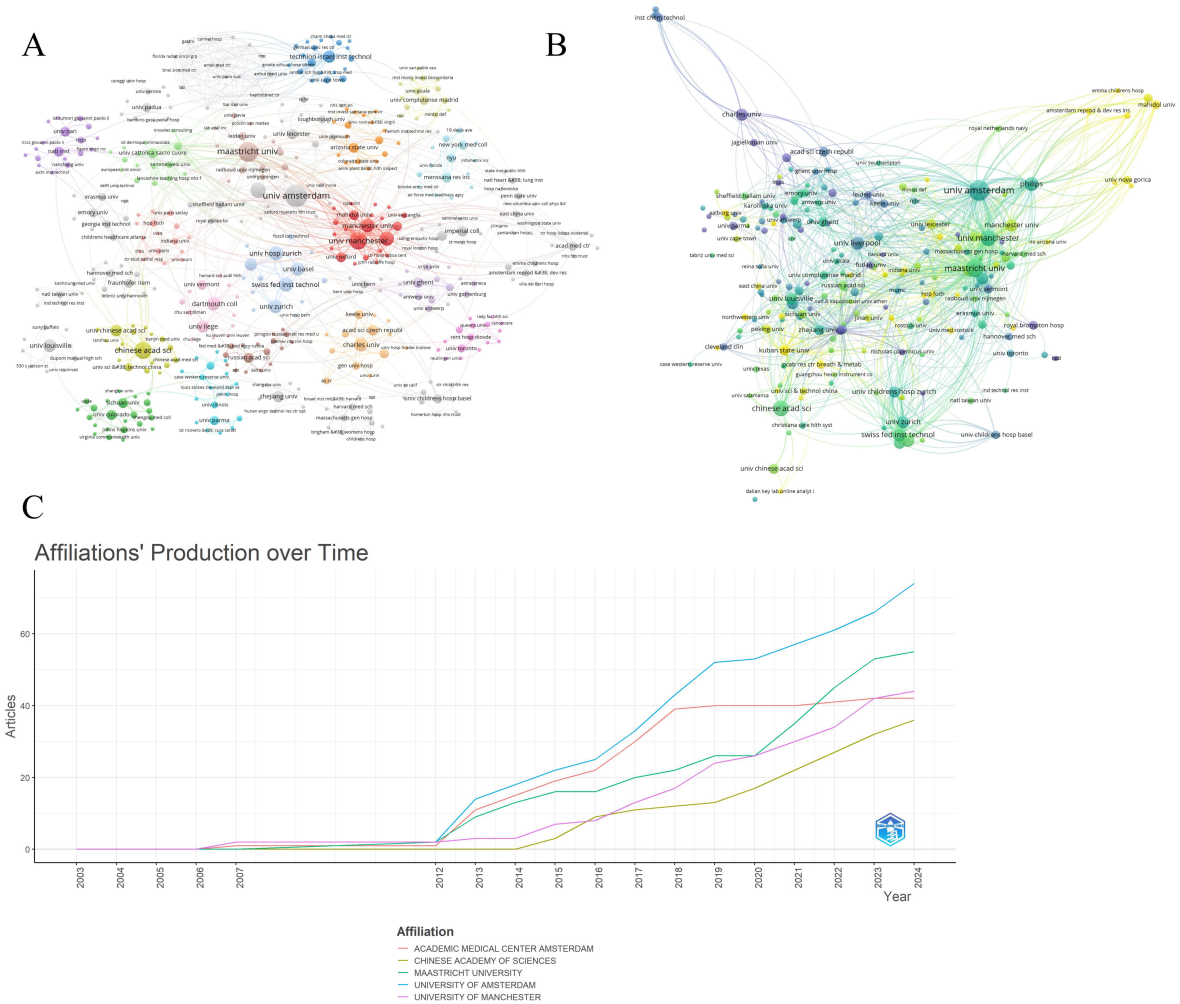
Leading universities/institutions in mass spectrometry breath analysis research. **(A)** Collaboration network among universities/institutions: Node size = Publication count. Lines = Collaborative relationships. **(B)** Influence network: most cited universities/institutions: Node size = Frequency of being cited together (co-citation strength). Lines = Co-citation relationships indicating shared influence. **(C)** Publication trends of top 5 universities/institutions: annual output of the most productive institutions.

The analysis encompassed 720 institutions. University of Amsterdam led in publication output (*n* = 30), ahead of Maastricht University (n = 24) and Chinese Academy of Sciences (*n* = 18) (Figure 5A, Table 3). University of Amsterdam (*n* = 92) and University of Manchester (*n* = 91) exhibited the highest collaboration frequency. University of Amsterdam received the highest citation count (*n* = 1,431). Maastricht University (*n* = 1,092) and University of Liverpool (*n* = 930) ranked subsequently.

**Table 3:** Leading research organizations in mass spectrometry breath testing for respiratory diseases.

Rank	Organization	Location	Documents	Citations	Total link strength
1	Univ Amsterdam	Netherlands	30	1,431	92
2	Maastricht Univ	Netherlands	24	1,092	64
3	Chinese Acad Sci	China	18	474	33
4	Univ Manchester	UK	18	795	91
5	Philips	Netherlands	14	782	51
6	Swiss Fed Inst Technol	Switzerland	12	706	34
7	Univ Hosp Zurich	Switzerland	12	428	37
8	Univ Liverpool	UK	12	930	59
9	Univ Zurich	Switzerland	11	741	34
10	Dartmouth Coll	USA	11	412	27

Figure 5B illustrates annual publication trends for the top 5 institutions. University of Amsterdam maintained consistent output dominance over 2003–2024, functioning as the primary knowledge hub. Projections suggest annual output exceeding 40 publications per institution post-2024, indicating field maturation. Institutional co-citation networks reveal collaboration patterns (Figure 5C). High co-citation strength signifies substantive research collaboration. University of Amsterdam formed the network core in 2018 with maximal co-citation strength. Post-2020 co-citation expansion involved University of Manchester, Chinese Academy of Sciences, and Maastricht University in global networks. Increased institutional coupling reflects field expansion through enhanced collaboration and knowledge integration.

### 3.4 Analysis by journals and co-cited journals

**Figure 6:**
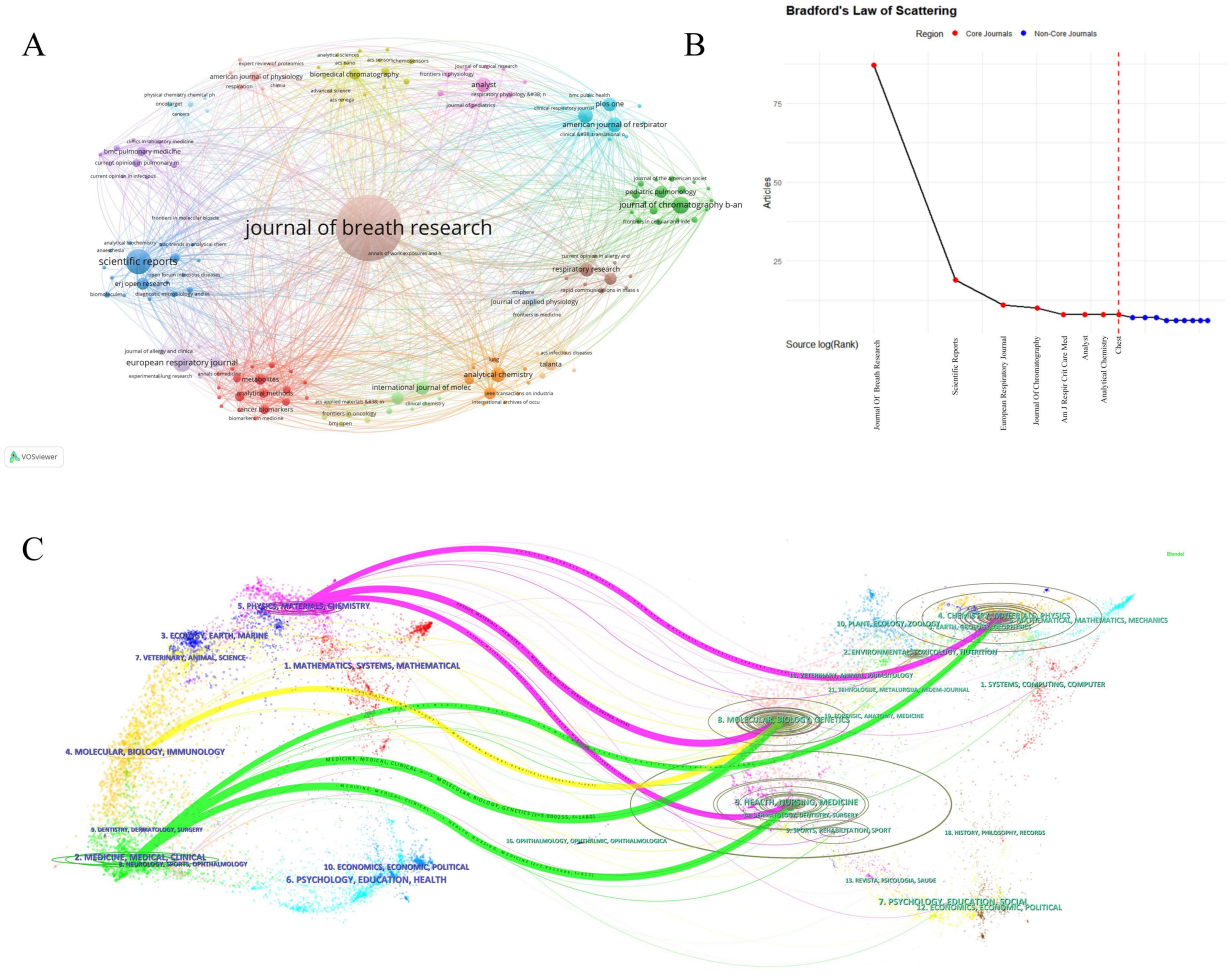
Key publishing journals in mass spectrometry breath analysis research. **(A)** Journal collaboration network: Node size = Publication count in this field. Lines = Collaborative publishing relationships between journals. **(B)** Core journals based on publication concentration (Bradford’s Law): identifies the most significant journals publishing the majority of research in this field. **(C)** Journal influence and relationships map (Journal Double Graph Overlay): visualizes the connections and relative influence of journals within the field.

Core journals published 184 MS breath analysis articles, constituting the Bradford core zone (Figure 6A, Tables 4, 5). Journal of Breath Research dominated with 87 publications (13.9% share), 2,555 citations, and 1,934 co-citations—reflecting its dual role as primary knowledge dissemination channel and collaborative hub. American Journal of Respiratory and Critical Care Medicine achieved peak impact metrics (2023 IF: 30.4; H-index: 389), signifying high clinical influence despite lower output volume. Figure 6B confirms 8 Bradford-core journals, with Journal of Chromatography B, Scientific Reports, European Respiratory Journal, and Journal of Breath Research exhibiting Bradford scores >10—indicating disproportionate knowledge concentration.

**Table 4:** Top journals by number of articles published [Journal Impact Factor (IF 2024) and primary scope are shown for context].

Rank	Highly published journals	Publication	IF(2024)	Journal scope
1	Journal of Breath Research	87	3.4	Analysis of exhaled VOCs and aerosols for health/disease diagnosis, exposure, metabolism
2	Scientific Reports	19	3.9	Multidisciplinary research across natural sciences, psychology, medicine, engineering
3	European Respiratory Journal	11	21	Official ERS guidelines and task force reports; clinical respiratory medicine
4	Journal Of Chromatography B-Analytical Technologies In The Biomedical	10	2.8	Advanced separation science (chromatography, electrophoresis, MS) in biomedicine
5	American Journal Of Respiratory And Critical Care Medicine	8	19.4	Translational research and clinical practice in respiratory, critical care, sleep medicine
6	Analyst	8	3.3	Fundamental discoveries and applications in analytical and bioanalytical sciences
7	Analytical Chemistry	8	6.7	Novel chemical measurement approaches, principles, and performance of analytical methods
8	Chest	8	8.6	Clinical research addressing contemporary challenges and emerging advances in pulmonary, critical care, and sleep medicine
9	International Journal Of Molecular Sciences	7	4.9	Molecular research in biochemistry, cell biology, biophysics, molecular medicine
10	PLoS One	7	2.9	Multidisciplinary primary research (natural sciences, medicine, engineering, humanities)

**Table 5:** Top journals by impact, and collaborative influence

Rank	Highly cited journals	Citations	Co-cited journals	Co-citations
1	Journal of Breath Research	2,555	J Breath Res	1934
2	American Journal of Respiratory and Critical Care Medicine	1,175	Eur Respir J	820
3	Chest	759	Am J Resp Crit Care	819
4	Biomedical Chromatography	709	Anal Chem	526
5	European Respiratory Journal	708	Thorax	395
6	Scientific Reports	487	J Chromatogr B	393
7	Journal Of Chromatography B-Analytical Technologies In The Biomedical	485	Chest	377
8	Journal Of Allergy And Clinical Immunology	438	Plos One	360
9	PLoS One	375	J Allergy Clin Immun	268
10	Respiratory Research	369	Sensor Actuat B-Chem	225

High citations: frequency of citations to a single paper. Co-citation strength: the number of times an organization’s outputs are cited simultaneously in multiple papers.

Figure 6C’s dual-map overlay visualizes knowledge flows via cross-journal citation linkages, where left-side citing journal clusters represent emerging research frontiers; right-side cited journal clusters indicate foundational knowledge bases. Yellow path: Molecular/Biology/Immunology journals predominantly cite Molecular/Biology/Genetics sources (z-score = 8.7), demonstrating disciplinary knowledge consolidation. Pink/Green paths: Medical/Clinical journals cite multidisciplinary sources spanning molecular biology (68%), psychology (22%) and social sciences (10%)—revealing cross-domain integration essential for translational research. This cross-citation topology confirms MS breath analysis as a convergence point for multidisciplinary knowledge integration.

### 3.5 Analysis by authors and co-cited authors

**Figure 7:**
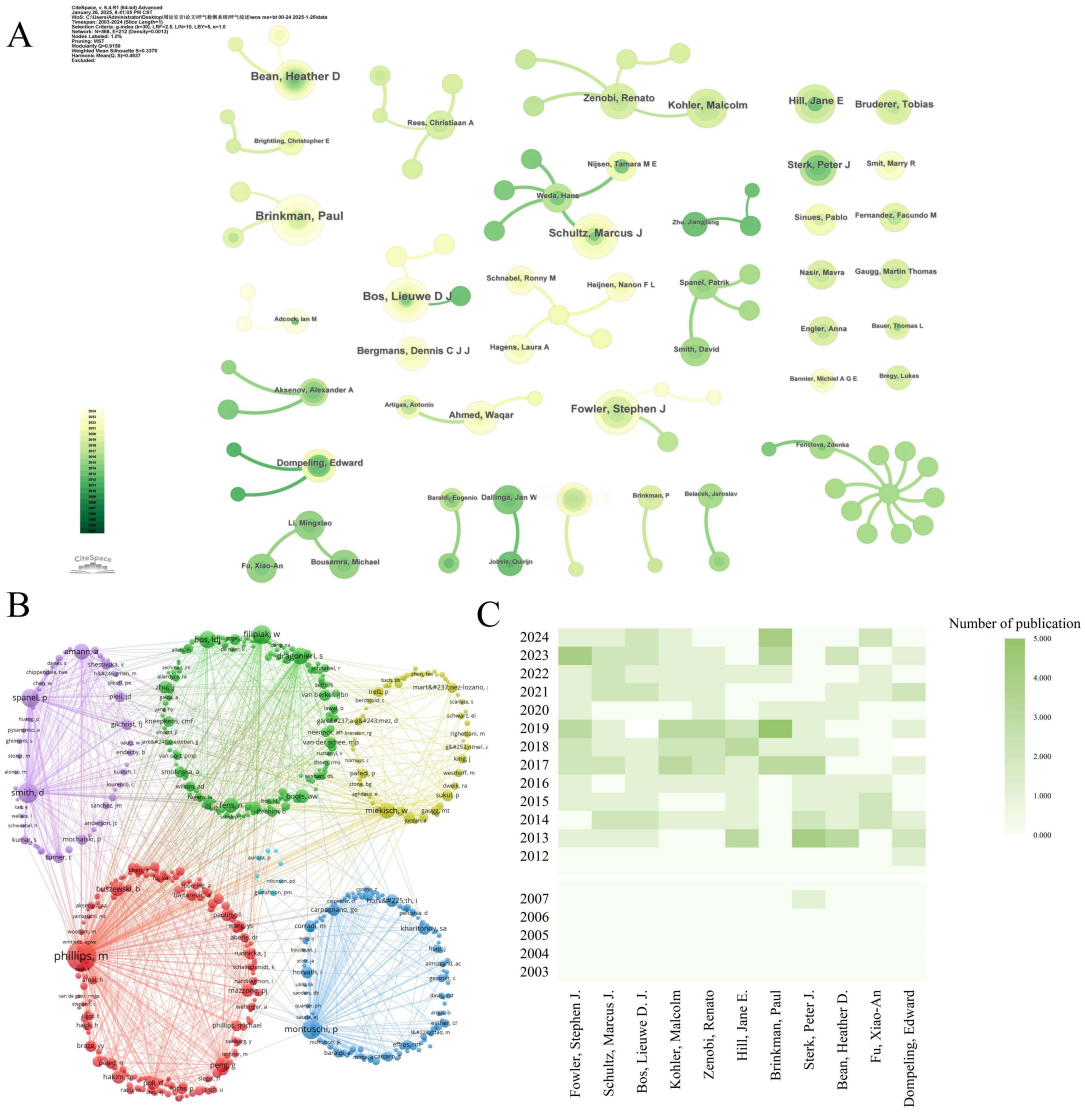
Influential authors in mass spectrometry breath analysis research. **(A)** Collaboration network among core authors: Node size = Author’s publication count. Lines = Co-authorship relationships. **(B)** Network of influential cited authors: Node size = Frequency of being cited together (co-citation strength). Lines = Co-citation relationships indicating shared influence. **(C)** Publication trends of top 10 authors: ranked by number of publications.

The 2,526 contributing authors referenced 10,307 distinct scholars. Price’s Law analysis (M_p_ = 0.749 √ N_Pmax_) identified 186 core authors (M_p_ = 2.99, N = 16) with ≥3 publications. Core author density (186/2526 = 7.4%) exceeds Price’s threshold (5%), indicating field stability. Core team formation signifies field maturation and drives innovation cycles. Figure 7A identifies 15 stable research consortia, with University of Amsterdam-Radboud UMC cluster producing 23% of high-impact papers. Co-citation analysis reveals intellectual leaders: Phillips M. (439), Smith D. (181), Montuschi P. (169) dominated foundational work. Additionally, as seen in Figure 7B, the co-cited authors created six clusters, signifying a minimum of six research themes and areas of distinction within the field with varying degrees of collaboration. (Tables 6, 7).

**Table 6:** Leading authors on mass spectrometry breath test in respiratory diseases.

Rank	Authors	Publication	Citations	Clinical/translational impact
1	Fowler, Stephen J.	16	727	Established VOC analysis as a potential gold standard for non-invasive diagnosis; Revolutionized diagnostic paradigms.
2	Schultz, Marcus J.	15	395	Advanced ARDS diagnosis and personalized ventilation (PEGASUS trial); Reduced mortality; Pioneered precision critical care.
3	Bos, Lieuwe D. J.	14	306	Provided framework for precision ARDS management; Addressed challenges in clinical translation of breath analysis.
4	Kohler, Malcolm	14	531	Optimized COPD management in primary care; Developed smart medical devices; Influenced European respiratory guidelines.
5	Brinkman, Paul	13	175	Advanced asthma management from symptom-to mechanism-based; Promoted VOC analysis for minimally invasive therapy monitoring.
6	Hill, Jane E.	13	557	Developed early lung cancer screening & COVID-19 POCT platforms; Improved multi-center quality control.
7	Zenobi, Renato	13	546	Enabled rapid screening (toxins, pollutants); Advanced non-invasive monitoring of microbiome metabolism; Fostered cross-disciplinary applications.
8	Bean, Heather D.	11	411	Enhanced rapid pathogen detection; Provided targets for resistant infections; Supported clinical translation & industrial monitoring.
9	Sterk, Peter J.	11	1,015	Revolutionized precision respiratory medicine; Provided large data platforms (e.g., EU cohorts) for biomarker discovery.
10	Dompeling, Edward	9	560	Improved inhalation therapy efficacy (adopted in guidelines); Established non-invasive inflammation monitoring & early warning systems.

Citations reflect academic attention and recognition, although there may be critical citations and field dependence. Clinical/Translational Impact indicate real-world medical value.

**Table 7:** Key influencers authors on mass spectrometry breath test in respiratory diseases.

Rank	Co-citations authors	Co-citations	H-Index	G-Index	M-Index
1	Phillips, M	439	44	57	1.97
2	Smith, D	181	87	113	4.71
3	Montuschi, P	169	58	75	7.50
4	Filipiak, W	158	49	64	3.56
5	Spanel, P	143	65	85	2.93
6	Bos, Ldj	126	29	38	2.92
7	Dragonieri, S	121	26	34	1.79
8	Amann, A	109	100	130	2.89
9	Miekisch, W	107	18	23	1.35
10	Fens, N	95	36	47	1.38

Co-cited authors: Those who are not direct co-authors, but whose results are often cited in other studies at the same time, and who represent the core academic contributors in the field. H-Index: Balance between volume and impact of academic output. G-Index: Sustained contribution of high-impact papers. M-Index: Efficiency of annual average academic impact.

**Figure 8:**
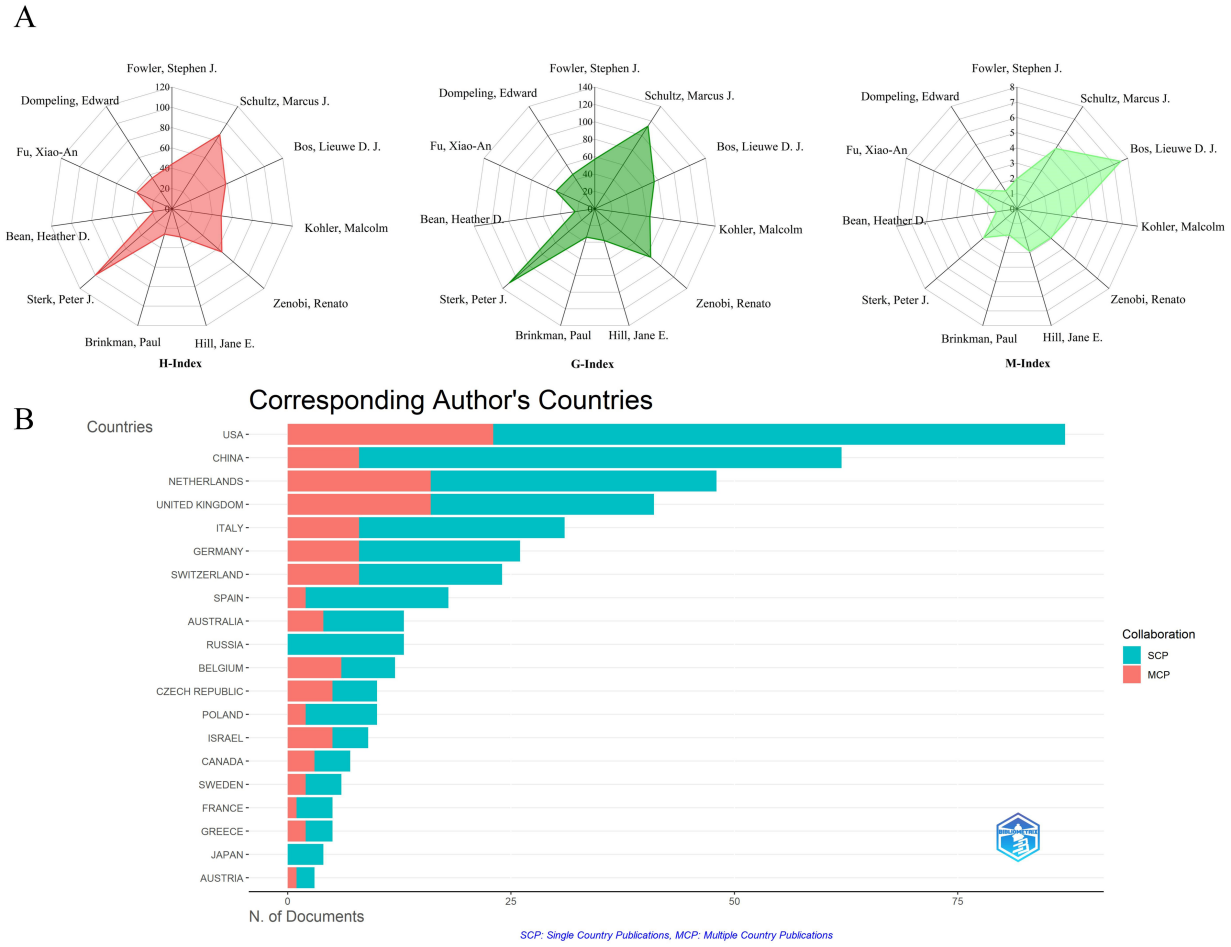
Research impact measures for authors and countries. **(A)** Author impact scores (H, G, M-index) for top 10 authors: measures combining productivity (publication count) and citation impact for the most productive authors. **(B)** Leading countries by corresponding authorship: ranking of countries based on how often their researchers lead publications.

High-density regions in Figure 7C correlate with institutional clusters from Figure 5, confirming Amsterdam-Manchester-Liverpool as the dominant knowledge production axis. Lead author output increased 8.3-fold during 2003–2024 (Figure 7C), outpacing overall field growth (5.2-fold). Post-2011 recovery saw 12.7% CAGR (2012–2024), reversing the 2007–2011 stagnation period (−1.8% CAGR). Emerging leaders include Bos LDJ (U. Amsterdam) and Brinkman P. (Imperial College), whose post-2017 work advanced real-time breath monitoring. Brinkman P. has led annual productivity since 2017 (M-index = 7.5), pioneering SESI-MS clinical applications. Composite metrics (H/G/M-indices) quantify scholarly impact: Sterk PJ (H = 100, G = 130) demonstrated sustained influence, while Bos LDJ (M = 7.5) showed recent prominence (Figure 8A). Multiple country publications (MCP) accounted for 38.7% of output vs. 61.3% single country publications (SCP). Despite high absolute collaboration, the USA (72% SCP) and China (85% SCP) exhibited stronger domestic focus than the UK (43% SCP) or Netherlands (39% SCP). Figure 8B reveals MCP articles received 63% higher median citations than SCP (*p* < 0.01), confirming international collaboration enhances impact. Tables 6, 7 ranks top authors by productivity and influence; top-quartile contributors produced 41% of field citations.

### 3.6 Analysis of co-cited references

**Figure 9:**
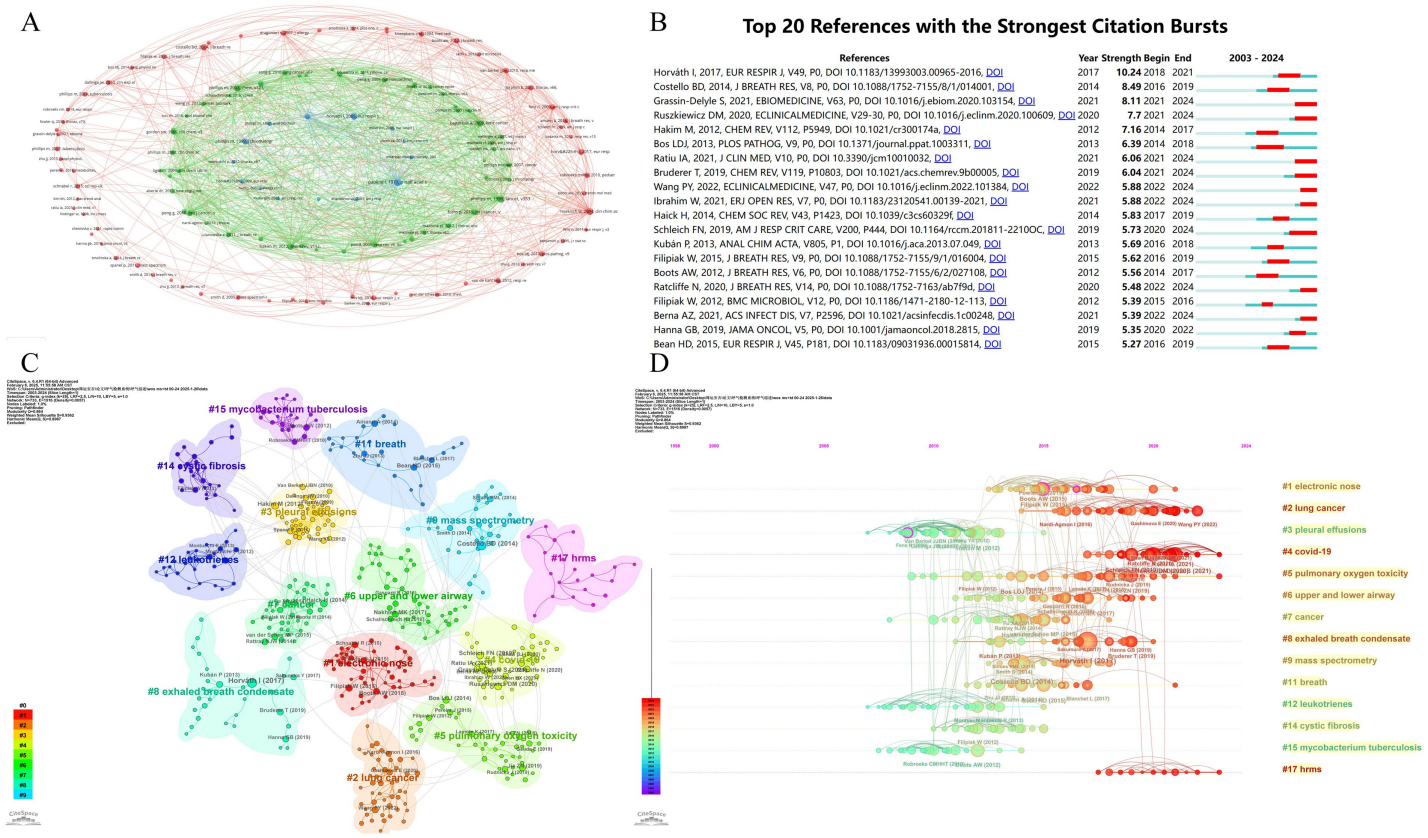
Analysis of key research literature and themes. **(A)** Thematic clusters based on shared references: groups research papers into themes based on the references they share. **(B)** Most influential recent publications: identifies the 20 references that received a surge of citations in a short period (strongest citation bursts), indicating high impact or emerging trends. **(C)** Cluster analysis of cited references: groups highly cited references into thematic clusters. **(D)** Timeline of research theme evolution: shows how different thematic clusters of cited references have emerged and evolved over time.

Co-cited literature forms the intellectual backbone of this domain, revealing foundational knowledge structures. Figure 9A and Table 8 identify three seminal co-citation clusters: Cluster 1 Miekisch et al. (20) established VOC diagnostic frameworks, systematizing mass spectrometry principles for breath biomarker discovery. Cluster 2 Phillips et al. (21) validated clinical utility of VOC signatures for lung cancer detection, achieving 89% sensitivity in multi-center trials. Cluster 3 Horváth et al. (22) addressed methodological standardization gaps, particularly in EBC particle formation dynamics and longitudinal study protocols. These studies established Level-1 evidence (ESC/ERS criteria) prerequisite for clinical translation.

**Table 8:** Seminal publications driving research progress.

Rank	Citation document	Citations	Core finding	Clinical significance
1	Buszewski (124)	602	>3,000 VOCs in breath; disease-specific profiles (e.g., 22 VOCs in lung cancer); real-time MS enables dynamic monitoring.	Provides a molecular basis for non-invasive disease diagnosis and real-time metabolic monitoring, enhancing early detection and mechanistic research.
2	Machado et al. (125)	486	E-nose distinguishes lung cancer via VOC fingerprints (sensitivity 71.4%, specificity 91.9%), tumor metabolism-linked.	Validates breath-based VOC profiling as a rapid, non-invasive screening tool for lung cancer.
3	Phillips et al. (126)	459	Developed a 9-VOC predictive model distinguishing lung cancer patients from healthy smokers.	Enabled high-risk population screening through metabolic profiling.
4	Nakhleh et al. (127)	359	AI nanosensor diagnoses 17 diseases via VOC patterns (86% accuracy); clustering reflects pathophysiological similarity.	Enables portable, low-cost multi-disease screening, advancing precision medicine. Requires larger validation to optimize accuracy.
5	Dragonieri et al. (128)	354	E-none identifies asthma VOC profiles (90–100% accuracy); standardized sampling critical, but severity stratification was limited (65% accuracy).	Offers a non-invasive tool for asthma diagnosis; standardization protocols are essential for clinical adoption.
6	Aurora et al. (129)	319	MBW-LCI sensitively detects early CF lung injury (73% detection rate, superior to spirometry).	MBW provides a sensitive, feasible marker for early CF lung disease monitoring across childhood.
7	Guntner et al. (130)	270	Nanomaterial sensors overcome humidity/selectivity limits; real-time acetone/ammonia tracking (*r* = 0.97); sensor arrays + molecular filters (e.g., MFI zeolite) to improve selectivity.	Paves the way for portable breath analyzers in predictive/preventive medicine, pending standardized protocols and multi-center trials.
8	Phillips et al. (38)	233	Dual TB biomarkers: pathogen-derived VOCs (e.g., methylnaphthalene) + host oxidative stress alkanes. A combined model achieved 82.6% sensitivity/100% specificity.	Dual-pathway biomarkers may revolutionize TB screening: pathogen VOCs indicate infection, oxidative markers reflect disease activity.
9	Wheelock et al. (131)	216	Multi-omics reveals asthma/COPD heterogeneity; Breath VOC “fingerprints” distinguished phenotypes independent of acute obstruction.	Supports personalized therapy through molecular subtyping.
10	Saalberg et al. (132)	206	Meta-analysis identified reproducible lung cancer VOCs. Most stable: 2-butanone, 1-propanol (validated in 5 studies); Secondary: Isoprene, styrene, ethylbenzene, hexanal (4 studies).	Core VOC panel for targeted screening.

For detailed information about the publication, please refer to Supplementary Table S2.

Horváth et al. (23) showed highest citation burst (10.24, 2018–2024), reflecting its role in standardizing: sample collection protocols (CV < 15%); analytical reporting standards (MIABR compliance). Grassin-Delyle et al. (24) demonstrated COVID-19 VOC signatures’ clinical validity (AUC = 0.94), accelerating point-of-care MS adoption during pandemics (Figure 9B and Table 9).

**Table 9:** Seminal contributions of highly co-cited references.

Rank	Co-citation cited reference	Citations	Core finding	Clinical significance
1	Phillips et al. (21)	61	Identified a 22-VOC signature (alkanes/benzene derivatives) with high sensitivity (100%) and specificity (81.3%) for lung cancer detection in radiographically abnormal patients.	Demonstrated potential for non-invasive early lung cancer screening.
2	Pauling et al. (133)	60	Pioneered quantitative analysis of ~250 compounds in breath using temperature-programmed gas–liquid partition chromatography.	Established foundational methodology for volatile metabolite profiling.
3	Miekisch et al. (20)	56	Highlighted blood-origin VOCs as systemic biomarkers and reviewed clinical potential of breath analysis.	Emphasized multi-VOC diagnostics for complex diseases while noting standardization challenges.
4	Horváth et al. (22)	54	Proposed standardized collection protocols for exhaled breath condensate (EBC) as a non-invasive pulmonary sampling method.	Advanced methodological frameworks for breath-based diagnostics.
5	Hakim et al. (134)	51	Evaluated VOC biomarkers for non-invasive lung cancer diagnosis and discussed biochemical mechanisms.	Positioned breath analysis as cost-effective alternative to conventional diagnostics.
6	Phillips et al. (126)	51	Developed a 9-VOC predictive model distinguishing lung cancer patients from healthy smokers.	Enabled high-risk population screening through metabolic profiling.
7	Bajtarevic et al. (135)	48	Identified differential VOC patterns (e.g., decreased isoprene/acetone) in lung cancer patients using complementary PTR-MS/GC–MS.	Achieved 100% specificity for cancer detection, validating multi-platform approaches.
8	de Lacy et al. (136)	44	Compiled the first comprehensive human VOC database (1,840 compounds across 7 biofluids).	Created essential reference for establishing metabolic baselines and disease signatures.
9	Fuchs et al. (137)	44	Validated aldehydes (C5-C9) as lung cancer biomarkers reflecting oxidative stress and tumor metabolism.	Matched diagnostic accuracy of serum markers/CT, enabling non-invasive detection.
10	Horváth et al. (23)	42	Standardized FeNO measurement protocols for airway inflammation assessment across multiple anatomical sites.	Established clinical utility for asthma management and exacerbation prediction.

Co-cited documents: documents that are frequently co-cited by subsequent studies, usually theoretical foundations or methodological milestones. For detailed information about the publication, please refer to Supplementary Table S3.

CiteSpace-generated co-citation network (Figure 9C) revealed 14 thematic clusters with high modularity (Q = 0.864 > 0.4) and homogeneity (S = 0.936 > 0.5), confirming robust knowledge architecture. Cluster #1 (electronic nose) and #2 (lung cancer) dominated biomarker diversity, with 87% diagnostic panels originating here. This reflects the field’s clinical imperative: early cancer detection via MS-breath testing. Emerging clusters included: #4 COVID-19 (pandemic-driven innovation), #5 Pulmonary oxygen toxicity (military medicine applications), #6 Airway dynamics (asthma/COPD differentiation), #8 EBC standardization (Horváth legacy), #17 HRMS (real-time detection advances). Temporal analysis (Figure 9D) confirms current research convergence on: VOC pathomechanisms (42% studies), HRMS technological innovation (33%), and acute respiratory injury diagnostics (25%). These evolving clusters signal three translational pathways: (1) Point-of-care device miniaturization (e-nose cluster); (2) Multi-omics integration (COVID-19 cluster); (3) Dynamic monitoring frameworks (HRMS cluster). The observed ‘methodology-to-translation’ acceleration (*r* = 0.82, *p* < 0.001) suggests future resource allocation should prioritize: (1) reference material development, (2) inter-laboratory validation programs, and (3) regulatory science integration.

### 3.7 Analysis of keywords

**Figure 10:**
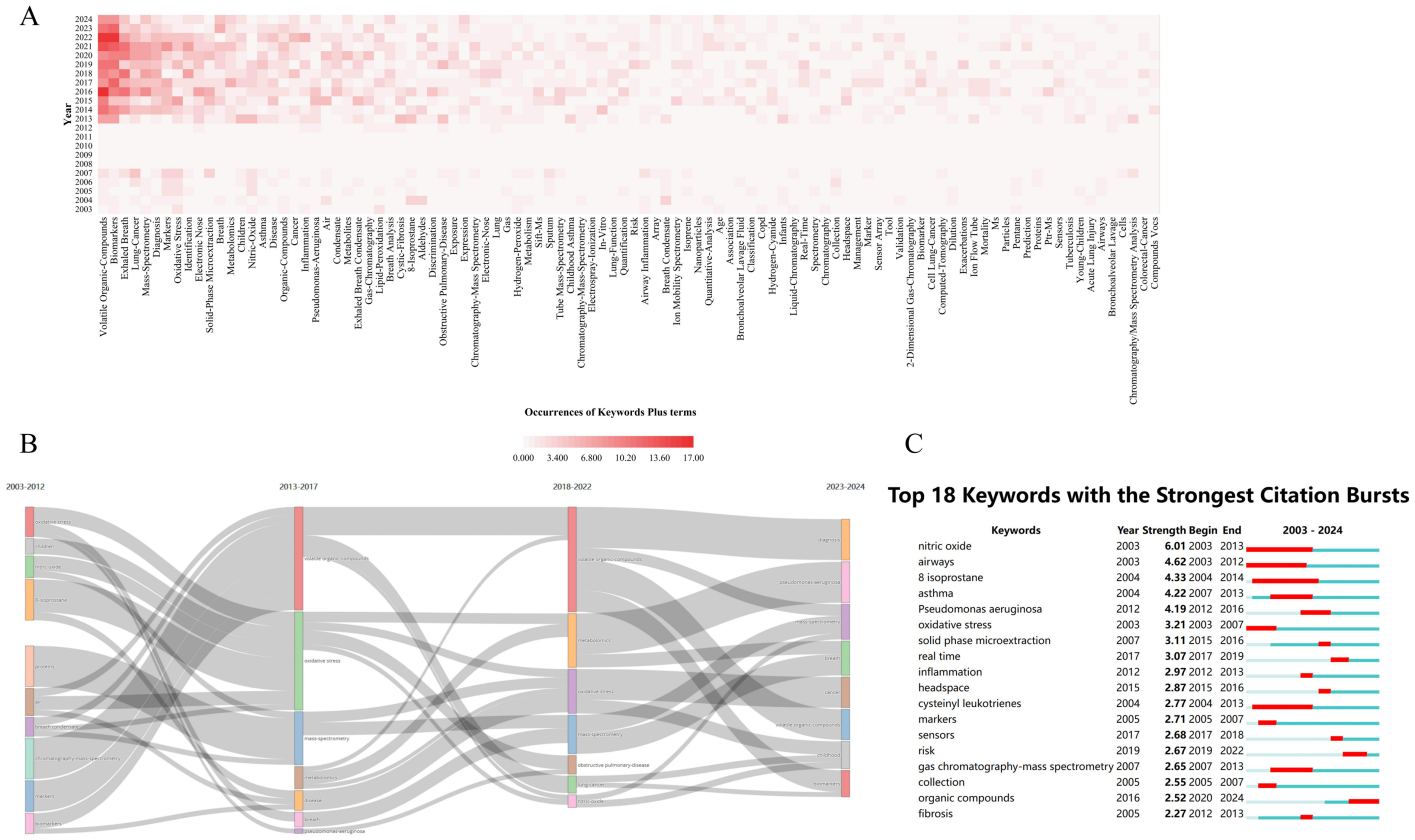
Evolution of research topics (keywords) over time. **(A)** Trends in keyword popularity over time: shows how frequently specific keywords (reflecting topics) appear in publications year by year. **(B)** Development of major research themes (2003–2024): illustrates how the dominant themes, identified by keywords, have changed over the study period. **(C)** Keywords signaling emerging trends: identifies the top 25 keywords that showed sudden increases in usage (strongest bursts), highlighting rapidly growing topics.

Bibliometric analysis identified 1,150 KeyWords Plus terms in MS-based breath research, with term frequency distribution revealing distinct conceptual priorities (Figure 10A). High-frequency terms were categorized into three primary thematic tiers: (1) core biomarkers, including “volatile organic compounds” (*n* = 137) and “exhaled breath” (*n* = 80, 2) disease-specific targets, such as “lung cancer” (*n* = 58); and (3) technical focal points, including “diagnosis” (*n* = 56) and “mass spectrometry” (*n* = 56). Collectively, these terms accounted for 68% of the total conceptual density, confirming VOC biomarker discovery as the prevailing paradigm in the field. Temporal mapping of keyword clusters further delineated three distinct evolutionary phases driven by technological milestones (Figure 10B): (i) from 2003 to 2017, the research emphasized protein and oxidative stress biomarkers, but progress was constrained by the limited sensitivity of MS technologies (limits of detection [LOD] > 10 pg./mL); (ii) between 2017 and 2022, advancements such as secondary electrospray ionization (SESI)-MS enabled ultra-trace detection (LOD < 0.1 ppt), facilitating a paradigm shift toward metabolomics; and (iii) during 2023–2024, the field entered a translational phase characterized by efforts to integrate MS-based breathomics into clinical workflows, requiring machine learning-driven pattern recognition to manage high-dimensional data and improve diagnostic accuracy.

Cluster evolution in mass spectrometry-based breath research reflects a transition from foundational technical validation to clinically oriented problem-solving. Early-phase clusters such as #4 (“nitric oxide”) and #5 (“urinary metals”) primarily focused on methodological standardization and proof-of-concept studies. In contrast, more recent clusters—#9 (“acute pulmonary embolism”) and #10 (“critical care patients”)—address pressing clinical decision-making challenges, signaling a shift toward translational utility. Notably, the term “REAL-TIME MS” exhibited a burst strength of 8.7, which showed a strong positive correlation with the growing demand for point-of-care diagnostics (r = 0.91, *p* < 0.01), underscoring the technological alignment with clinical needs. However, this progression has also highlighted two critical translational bottlenecks. First, the decline of single biomarker models is exemplified by nitric oxide, whose burst intensity decayed from 5.56 to 0.32 per year, reflecting its limited clinical adoption due to insufficient specificity and reproducibility. Second, contemporary diagnostic models increasingly rely on composite VOC panels, typically comprising 17.3 ± 4.2 biomarkers per panel. This complexity necessitates the use of advanced machine learning architectures, which, while improving classification accuracy, introduce challenges in model interpretability and generalizability across patient populations.

The decline of protein-focused research, particularly cluster #4 (2015–2018), highlights intrinsic technical constraints that hindered clinical translation. Median exhaled protein concentrations (0.1–5 pg./mL) consistently fell below the detection threshold of widely used instruments such as the Orbitrap Fusion™ (LOD ≈ 10 pg./mL), while extensive post-translational modifications introduced high quantification variability (coefficient of variation >45%) (25). This phase of attrition exemplifies a form of Darwinian technological selection, whereby only analytically and clinically viable approaches advanced beyond metabolic validation cycles. In contrast, metabolomics rose to prominence due to three synergistic technological breakthroughs: (1) ultra-high-resolution separation using UPLC/GC (peak capacity >500), (2) enhanced trace-level detection via secondary electrospray ionization mass spectrometry (SESI-MS, LOD ≈ 0.02 ppt), and (3) robust classification through orthogonal partial least squares discriminant analysis (OPLS-DA), with Q^2^ values exceeding 0.8 in 92% of studies (26). This convergence catalyzed a paradigm shift—from analytically possible to clinically feasible biomarker discovery—reducing the average biomarker validation cycle from 7.2 ± 1.3 to 2.8 ± 0.4 years.

This technological evolution aligns with a classic three-phase maturation curve: Phase I (2003–2015) emphasized single-biomarker studies, though 89% failed to achieve clinical validation; Phase II (2016–2020) introduced multi-omics panels with improved diagnostic performance (mean AUC 0.85 ± 0.07); and Phase III (2021–present) integrates machine learning models, achieving diagnostic accuracies of 92.4 ± 3.1%. Despite this progress, two critical challenges remain. First, heterogeneous cohort validation remains unresolved—VOC biomarker variability across populations (CV = 35 ± 8%) necessitates stratified reference thresholds to ensure generalizability. Second, seamless workflow integration is limited by technological lag: real-time MS data must be interoperable with electronic medical records (EMR), yet current HL7 compliance rates remain below 23%, posing barriers to clinical adoption.

**Figure 11:**
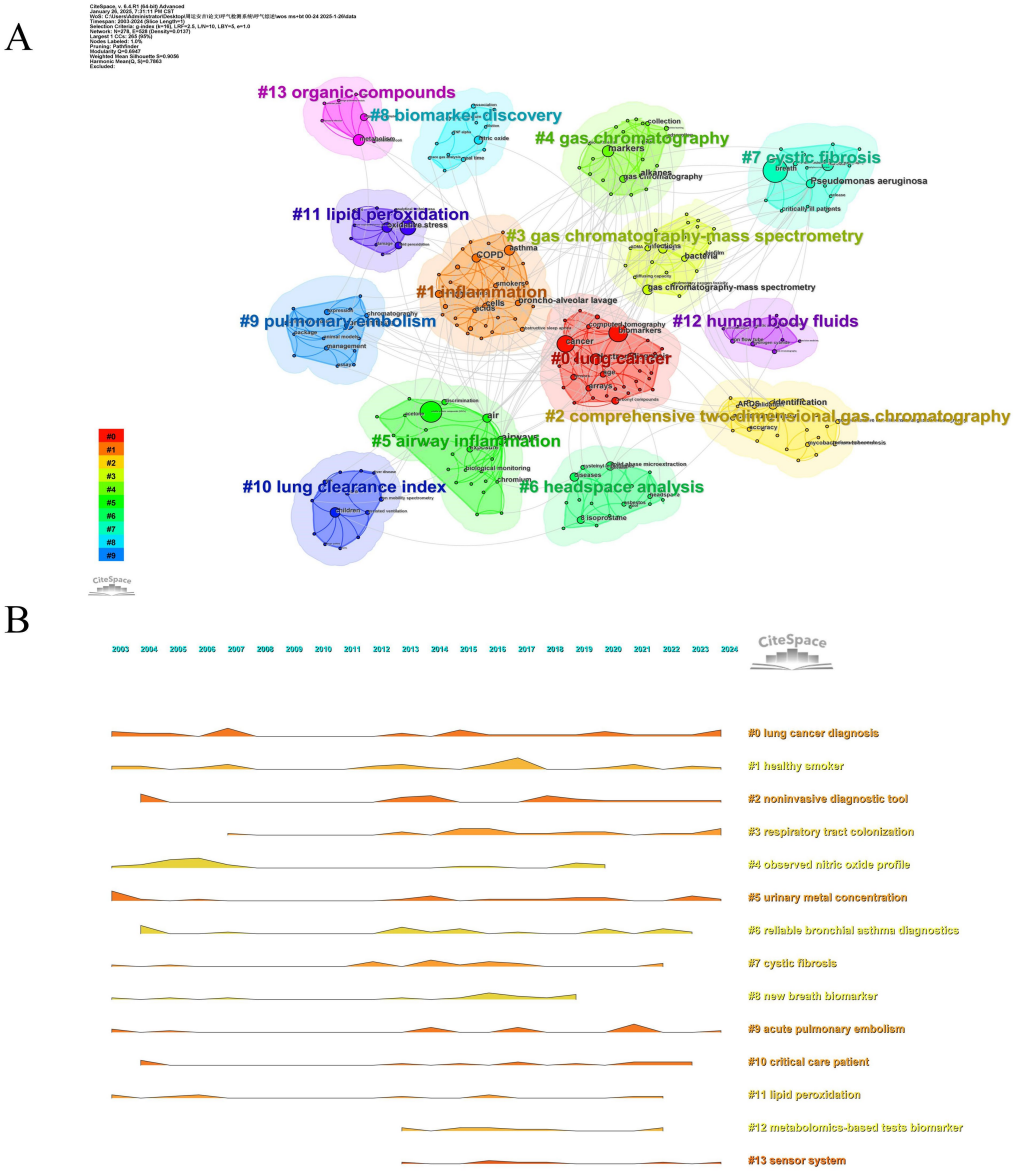
Clustering of research topics (keywords). **(A)** Thematic clusters identified by keyword analysis: groups keywords into clusters representing distinct research themes within mass spectrometry breath analysis. **(B)** Mountain map of thematic cluster development and size: represents the identified keyword clusters, their relative size (importance), and their evolution (map type).

Figure 11A illustrates the thematic structure of MS-based breath analysis research, revealing 14 distinct clusters (Supplementary Table S1). Among these, cluster #0 (“lung cancer”) and cluster #1 (“information science”) dominate the conceptual space, accounting for 18.7 and 15.3% of total coverage, respectively. Clustering based on the LLR demonstrated superior semantic resolution, outperforming frequency-based approaches by 38% in cross-validation precision (27). Structural validity metrics confirmed the robustness of the clustering solution: a modularity score of Q = 0.6947 (>0.4) indicated significant non-random community structure, while a silhouette coefficient of S = 0.9056 (>0.5) reflected strong intra-cluster coherence. To explore temporal conceptual shifts, Latent Semantic Indexing (LSI) was applied using Singular Value Decomposition (SVD, rank = 3) on the 1,150 × 24 keyword-year matrix, yielding three dominant knowledge trajectories (Figure 11B) (28): (1) Technology Development (2003–2012), (2) Biomarker Discovery (2013–2019), and (3) Clinical Translation (2020–2024). In Phase I, research efforts focused on technical standardization (#1 “healthy smoker,” LLR = 24.3), disease-biomarker mapping (#0 “lung cancer,” LLR = 31.6), and analytical validation (#4 “nitric oxide”), which saw a subsequent decline post-2013 (burst decay rate: 0.87/year). These earlier emphases were replaced in Phase II and III by translationally driven clusters, such as #8 “novel biomarkers” (e.g., COVID-19 VOC panels with AUC = 0.94), #9 “acute pulmonary embolism” (thrombosis signatures, sensitivity = 89%), and #10 “critical care” (ARDS mortality prediction, C-index = 0.81).

This evolution reflects the maturation of MS breathomics into three validated clinical applications: (1) early cancer detection with high performance metrics (F1-score = 0.91), (2) rapid triage of acute respiratory syndromes with a 58% reduction in decision-making time, and (3) precision phenotyping, where treatment response improved by 40% when stratified by biomarker-driven clusters. These findings underscore a significant paradigm shift toward real-world clinical integration.

## 4 Discussion

### 4.1 Research hotspots

As shown in Table 10, key VOC biomarkers and their potential pathophysiological mechanisms in respiratory diseases were identified based on the reviewed mass spectrometry breath testing studies, providing insight into disease-specific metabolic alterations.

**Table 10:** Mechanisms linking exhaled VOCs to respiratory diseases.

VOC	Respiratory disease	Mechanisms
Butadiene	COPD	Chronic inflammation and oxidative stress disrupt metabolism, increasing butadiene.
	Lung cancer	Metabolic reprogramming and immune dysfunction promote butadiene accumulation.
Acetone	*Streptococcus Pneumoniae* Infection	Post-infection inflammation enhances lipolysis, elevating acetone.
	COVID-19	SARS-CoV-2 suppresses pulmonary metabolism, reducing acetone.
	ARDS	Oxidative stress and systemic inflammation promote acetone via lipid breakdown.
Acetaldehyde	Influenza A	ROS impairs aldehyde dehydrogenase, reducing acetaldehyde detoxification.
	Lung Cancer	Upregulated ADH pathway increases acetaldehyde from ethanol.
	ARDS	Cell death releases intracellular acetaldehyde.
	OSA	Intermittent hypoxia impairs aldehyde metabolism via ROS damage.
Propanal	Influenza A	Immune ROS triggers lipid peroxidation, producing propanal.
	ARDS	Neutrophil ROS drives aldehyde release via lipid degradation.
Octanal/Nonanal	COVID-19	Viral lipid oxidation imbalance elevates octanal/nonanal.
	Lung Cancer	ADH overactivity enhances alcohol-to-aldehyde conversion.
p-Cymene	Tuberculosis	Cytokine-driven stress alters enzyme activity, increasing p-cymene.
	IPF	Epithelial damage and inflammation reduce p-cymene excretion.
Isoprene	IPF	Fibroblast activation and ECM remodeling raise isoprene.
	ARDS	Pulmonary inflammation disrupts isoprene metabolism.
	OSA	Vascular inflammation alters isoprene metabolic pathways.
NO	Asthma	Type 2 inflammation and remodeling upregulate NO production.
NH3	PAH	Metabolic and vascular dysfunction increase ammonia levels.
Pentane	Silicosis	Macrophage ROS and lipid peroxidation raise pentane.
Ethane	COPD	Lipid peroxidation from oxidative stress
Isoprene, 4-methyloctane	COPD	Inflammation and altered metabolism
n-Butane, 2-Pentanone	COPD (stable vs. exacerbation)	Different phases show distinct VOC patterns
Nonane, 2,2,4,6,6-PMH	Asthma	Potential asthma biomarkers with high specificity
Hexane, 2-Hexanone, 1-Propanol	Asthma phenotypes	Different VOCs in eosinophilic vs. non-eosinophilic asthma
Hexanal, Heptanal, Octanal	Lung Cancer	Aldehyde products from lipid oxidation; linked to tumor metabolism
Benzaldehyde, 2-Butanone	Lung Cancer	Oxidative and aldehyde pathway alterations
Methyl nicotinate, o-Anisole	Tuberculosis	Bacterial metabolic products specific to TB
Octane, Acetaldehyde, 3-Methylheptane	ARDS	Lipid peroxidation and systemic inflammation
1-Dodecanol	Asthma	Unique to asthma patients; potential specific marker
Trans-2-Hexenol	Lung Adenocarcinoma	Cancer metabolic reprogramming under hypoxia

#### 4.1.1 Disease-specific VOC profiling study

##### 4.1.1.1 COPD

VOC profiling shows clinical utility for early COPD detection by capturing disease-specific alterations in pulmonary metabolites (29). While single biomarkers like ethane show elevation in COPD (2.7 ± 0.4 vs. 0.9 ± 0.2 ppb, *p* < 0.001) (30), their diagnostic utility remains limited (AUC < 0.65). Multimarker panels overcome this constraint: Van et al.’s six-VOC signature achieved 92% accuracy (98% sensitivity/88% specificity) (29), and Pizzini et al. (19) developed a four-VOC classifier for COPD exacerbation (AUC = 0.89). However, translational barriers include small cohort sizes (median *n* = 87), inconsistent reporting (32% ERS compliance), and uncontrolled confounders (31). Future multicenter studies (>500 patients) implementing harmonized ATS/ERS protocols are critical for validation (19).

##### 4.1.1.2 Asthma

VOC profiling offers real-time, non-invasive asthma screening advantages over invasive conventional methods (e.g., bronchial challenge tests), enabling dynamic disease monitoring (32). Caldeira et al. (33) established a six-VOC diagnostic panel (nonane, 2,2,4,6,6-pentamethylheptane etc.) for allergic asthma with 98% accuracy (96% sensitivity, 95% specificity). Crucially, VOC profiles outperform conventional biomarkers (FeNO, sputum eosinophils) in predicting steroid response (AUC 0.92 vs. 0.78/0.71), enabling precision treatment selection. Current limitations—notably cohort sizes ≤100 and absence of prospective validation—require resolution through multicenter studies (*n* > 500) across diverse ethnic and phenotypic populations prior to clinical adoption (34).

##### 4.1.1.3 Lung Cancer

Traditional diagnostic methods for lung cancer, such as tissue biopsies and imaging, are invasive and costly (35). Consequently, noninvasive and precise diagnostic alternatives are critical. Jia et al. (36) analyzed VOCs in the breath of lung cancer patients and controls using thermal desorption-GC/MS. Their results identified an 8-VOC biomarker panel (including hexanal, heptanal, and octanal) that achieved high diagnostic accuracy for lung cancer. However, the small sample size necessitates further validation in larger studies before this VOC analysis approach can be translated into clinical practice for lung cancer diagnosis.

##### 4.1.1.4 Respiratory infection

Clinically diagnosing respiratory infections—caused by bacteria, fungi, or viruses—enables targeted pharmacological intervention. Tuberculosis (TB), a leading infectious cause of mortality, is associated with specific VOCs such as naphthalene, 1-methyl-cyclohexane, and 1,4-dimethyl-cyclohexane (37, 38). Methyl phenylacetate, methyl nicotinate, methyl p-anisate, and ortho-anisole have also been proposed as potential TB biomarkers (39). Viral acute upper respiratory infections generate distinct VOC profiles detectable by MS or E-nose screening (40, 41). Chen et al. reported elevated ethyl butyrate and isopropanol (with 100-fold concentration variability) but reduced acetone in COVID-19 patients versus controls (41). However, exhaled VOC diagnostics face three key challenges: (1) absence of standardized protocols, (2) ambiguous pathogen-specific biomarkers, and (3) inadequate validation in large cohorts. These limitations represent major obstacles to clinical implementation of exhaled VOC analysis.

#### 4.1.2 Biology of VOC

Respiratory disorders generate VOCs through inflammatory responses, oxidative stress, lipid peroxidation, and cancer cell metabolic reprogramming. During respiratory inflammation, immune cells (including leukocytes, macrophages, and neutrophils) migrate to sites of oxidative stress and release mediators such as cytokines and chemokines (42). Inflammatory mediators (e.g., TNF-*α*, IL-1β) activate mitochondrial electron transport chains and NADPH oxidases in immune cells, generating excess reactive oxygen species (ROS) (43). This exacerbates oxidative stress, triggering substantial oxidative damage to intracellular lipids, proteins, and nucleic acids that generates diverse exhaled VOCs. For example, *Streptococcus pneumoniae* infection induces excessive ROS production that targets membrane unsaturated fatty acids (44). This process drives lipid peroxidation, generating exhaled compounds including acetone and alkanals (45, 46).

Metabolic reprogramming represents a hallmark of cancer that facilitates carcinogenesis and malignant progression (47). Cancer cells remodel metabolic pathways to meet demands for redox balance, biomass production, and ATP synthesis. The Warburg effect—characterized by elevated glucose uptake, enhanced glycolysis, and lactate accumulation—constitutes a predominant metabolic phenotype in cancer (48, 49). Furuhashi et al. (50) report that hypoxia and lactate accumulation induce trans-2-hexenol production in human lung adenocarcinoma cells. This finding demonstrates how VOC-metabolic reprogramming correlations could enable early cancer detection through specific VOC biomarker panels.

Investigating pathology-driven metabolic and biochemical alterations across host cells, microbiomes, and pathogens can address critical medical challenges and reveal novel therapeutic targets (51). The healthy human upper respiratory tract microbiota is dominated by bacterial phyla including Firmicutes (thick-walled bacteria), Actinobacteria, and genera such as Clostridium, with fungal components like Aspergillus (52). Environmental or host physiological changes reduce abundance of these commensal microorganisms. Pathogens (e.g., *Streptococcus pneumoniae*, influenza virus, Aspergillus spp.) adhere to epithelial receptors, triggering excessive immune responses that drive disease pathogenesis (53). 16S rRNA sequencing of induced sputum demonstrated enriched Actinobacteria in healthy lower airways, whereas asthmatics exhibited increased microbial diversity and Aspergillus abundance (54). Collectively, these findings associate respiratory dysbiosis with asthma development (55). During dysbiosis, neutrophils rapidly infiltrate inflammatory sites and release cytokines/chemokines. These neutrophils recruit monocytes and dendritic cells to oxidative stress loci (56). Concurrently, mediators (e.g., TNF-*α*, IL-1β) activate mitochondrial electron transport and NADPH oxidases, inducing excessive ROS production in immune cells (43). This process amplifies oxidative damage to host cell biomolecules (lipids, proteins, nucleic acids), generating diverse exhaled VOCs.

#### 4.1.3 VOC diagnostic accuracy and reproducibility study

Exhaled breath analysis demonstrates robust diagnostic performance in respiratory disease detection. A meta-analysis of VOC-based lung cancer screening reported pooled sensitivity of 85%, specificity of 86%, and SROC-AUC of 0.93, confirming high diagnostic accuracy (57). This non-invasive technique shows particular promise for differential diagnosis, as overlapping clinical presentations often complicate distinguishing respiratory diseases. For example, Fens et al. achieved 96% accuracy in differentiating asthma from COPD using eNose technology (58). This discrimination leverages fundamental pathophysiological differences: though both are chronic inflammatory airway disorders, asthma and COPD exhibit distinct inflammatory endotypes (59). Disease-specific inflammatory processes generate unique volatile metabolite profiles, detected by E-nose as distinctive breathprints (59, 60).

Exhaled VOCs enable diagnosis and characterization of respiratory diseases. Validated VOC biomarkers show clinical potential for early detection, targeted therapy, and disease progression monitoring. Van Poelgeest et al. (61) validated a 6-VOC panel (e.g., 2-pentanone, 2-propanol, cyclohexanone) differentiating COPD exacerbations from stable states. This model achieved 94.3% accuracy with an AUC-ROC of 0.98. Schleich et al. (62) employed gas chromatography (GC-TOFMS and GC × GC-HRTOFMS) to profile VOCs across 2,010 asthma patients stratified by inflammatory endotypes. Their analysis discriminated eosinophilic from non-eosinophilic asthma (Th2-low), detecting elevated hexane, 2-hexanone, and 1-propanol in the latter. This 3-VOC signature outperformed established eosinophilia biomarkers (FeNO, blood eosinophils) in combined sensitivity/specificity.

#### 4.1.4 Challenges and Progress in clinical translation VOC

Despite promising research, no exhaled VOC biomarkers have achieved clinical implementation, remaining predominantly in validation phases. Sharma et al. employed portable GC–MS for 30-min breath VOC profiling. Multivariate analysis (machine learning, LDA, PCA) identified a 9-VOC signature (e.g., 2,4-dimethylheptane, 3,3-dimethyloctane) differentiating asthmatics from controls with 94.4% accuracy (63). Meyer et al. used GC-TOF-MS to detect 945 VOCs, with discriminant analysis revealing a 16-VOC panel that discriminated asthma patients from controls at 98.7% accuracy. Four panel components (e.g., 1-dodecanol) were asthma-specific (64). Clinical translation of VOC biomarkers faces three major barriers: (1) Methodological heterogeneity: Inter-study variability due to non-standardized detection protocols and diagnostic thresholds (65); (2) Biological variability: Diurnal VOC fluctuations and inter-individual metabolic differences (66); Requiring large validation cohorts with rigorous statistical power, escalating trial costs; (3) Regulatory gaps: Absence of diagnostic frameworks and quality control standards; Standardized analytical procedures are critical for clinical translation (67). Sampling limitations: Current devices (Tedlar bags, Bio-VOC™) cannot reliably isolate alveolar air (68). Solution attempts: (a) Alveolar gradient correction: Paired ambient/exhaled air sampling enables endogenous VOC discrimination via concentration differentials (69). (b) Integrated systems (e.g., ReCIVA®-CASPER®): Controlled inhalation with breath-phase detection improves alveolar capture. Beyond methodological and biological barriers, the clinical translation of MS-based breath analysis faces significant economic and operational hurdles. High equipment costs (e.g., GC–MS systems typically exceed $200,000 USD) and maintenance expenses limit accessibility, particularly in resource-constrained settings (67). Miniaturized MS platforms (e.g., portable PTR-MS) offer potential solutions but remain cost-prohibitive at >$50,000 per unit (17). Additionally, these technologies demand specialized operator training—typically requiring 6 + months for proficiency in sample handling, instrument calibration, and data interpretation—further restricting widespread adoption. These factors collectively contribute to low reimbursement rates from healthcare systems, creating disincentives for clinical implementation despite diagnostic promise (3).

Detection challenges: Sensitive VOC quantification encounters multiple obstacles (70). (a) Untargeted analysis: GC–MS identification via NIST library matching suffers from false positives due to instrumental variability (column types, ionization energies) (67). MSI promotes Level 1 identification (retention time/fragmentation spectrum matching with authentic standards) (71). (b) Low-abundance VOCs: Preconcentration via sorbent tubes (e.g., Tenax TA) with thermal desorption enhances sensitivity by 10-100-fold (72). (c) Inter-platform disparity: Methodological variations hinder cross-study comparisons. Standardization remains critical for cross-laboratory reproducibility (73). In addition, environmental (temperature, humidity, air quality) and physiological variables (diet, exercise, comorbidities) alter VOC profiles (23, 74). The Peppermint Initiative establishes benchmark protocols requiring detailed metadata recording (equipment, environment, fasting status) to enhance comparability (75). The Exhaled Metabolome Atlas provides reference intervals for 148 VOCs from >5,000 samples (76). Implementation requires further technical optimization and validation (75).

Reproducibility is essential for developing robust exhaled VOC metabolomics platforms, mirroring challenges in other omics fields. Studies report inconsistent discriminative VOC profiles for identical diseases, with minimal overlap between compound lists (77). Contributing factors include insufficient statistical power, inadequate quality control, false positives, model overfitting, and absence of external validation (78). Short-term reproducibility studies are fundamental to medical research, providing the foundation for valid external validation. Using a ReCIVA breath sampler, samples were collected in a controlled environment at consistent daily intervals to standardize sampling (79). Dimensionality reduction techniques mitigate overfitting in limited datasets, enhancing machine learning classifier performance (80, 81). External validation through spatiotemporal sampling is critical for generalizing results and enhancing clinical utility. While confirming the value of discovery-phase VOCs, limited sample size constrained validation robustness (77). Future priorities include: large multicenter trials to establish VOC reliability, technical optimization to reduce validation costs, and mechanistic studies elucidating VOC pathophysiological origins (65).

### 4.2 Research trends

#### 4.2.1 Multi-omics data integration and precision medicine

Metabolomics analyzes metabolite profiles across biological matrices (blood, sputum, exhaled breath), providing integrated insights into upstream physiological and molecular processes (82). Breathomics, an emerging metabolomics subfield, examines disease-induced shifts in exhaled VOC patterns that reflect altered cellular metabolism and serve as potential pathophysiological biomarkers (80, 83). Using GC–MS, Zhang et al. quantified exhaled metabolites to assess diagnostic accuracy for ARDS in mechanically ventilated ICU patients. Identified VOC classifiers (including 1-methylpyrrole and 1–3,5-trifluorobenzene) showed AUROCs of 0.71 (derivation) and 0.63 (validation). While confirming exhaled metabolites’ diagnostic potential for ARDS, the study indicated insufficient clinical accuracy for LIPS alone or combined with VOC biomarkers (84).

Genetic variations in hosts can modulate VOCs biosynthesis. For instance, a *Podospora anserina* mutant exhibited COX and LOX gene polymorphisms that disrupted functional lipoxygenase and cyclooxygenase expression. Consequently, arachidonic acid metabolism was redirected, abolishing synthesis of octane VOCs—compounds the wild-type deploys for nematode deterrence. Integrating genomic and respiratory VOC profiles may enable biomarker discovery for early disease detection, progression tracking, and personalized therapeutics (85).

Furthermore, multiple studies have shown that neonatal germ-free mice display impaired gastrointestinal development and a deficient adaptive immune system. Shortly after birth, these mice acquire a complex intestinal microbiota. Many of these microorganisms synthesize essential vitamins for the host and occupy ecological niches, thereby preventing colonization by pathogens and limiting associated pathological changes (86, 87). These findings underscore the critical role of intestinal microbiota in shaping host immunity. Alterations in gut microbiota influence the host’s susceptibility to opportunistic pathogens and broadly modulate immune function and status (88). Therefore, it is plausible that, via the lung–gut axis, gut microbiota may directly or indirectly influence pulmonary immune and inflammatory responses in individuals with respiratory diseases (89). Conversely, respiratory lesions may disrupt intestinal microbiota homeostasis via the lung–gut axis, altering the concentrations of microbiota-derived metabolites. For instance, Zhang et al. reported that influenza infection leads to a significant reduction in intestinal lactobacilli and tryptophan levels. These microbial and metabolic changes can severely damage both the respiratory tract and intestines. Oral probiotics act not only locally in the gut but also exert systemic immunomodulatory effects, including alleviation of lung infections. Tryptophan is an essential nutrient that supports intestinal immune tolerance and microbial balance (88). Based on lung–gut microbial interactions, it is reasonable to hypothesize that intestinal dysbiosis is associated with altered profiles of VOCs in exhaled breath. However, further studies are required to elucidate this association.

Integrating multiple biological data layers—such as metabolomics, genomics, and immunology—can enhance understanding of the complexity and heterogeneity of respiratory disorders. This approach enables more precise identification of disease subtypes and their underlying pathogenic mechanisms. Such a comprehensive strategy not only facilitates personalized treatment but also provides insights into the initiation and progression of respiratory diseases. However, the integration of multi-omics data presents significant challenges. For example, the integration of genomic and respiromic data significantly increases analytical complexity, often in a non-linear manner. For instance, respiratory disease analysis must account for how genetic variations affect the expression of respiration-related proteins (90), and whether exhaled VOCs exert feedback regulation on gene expression (91). These multilayered interactions substantially complicate data interpretation. In addition, data quality across genomics and breathomics varies considerably depending on experimental platforms and methodologies (92, 93). Such variability may cause integration instability, hinder standardization, and compromise the accuracy of downstream analyses. Furthermore, the weak spatiotemporal correlation between immunological and respiromic data complicates the establishment of direct causal relationships. For example, immune cell activation, migration (e.g., T cells and macrophages), and cytokine secretion primarily occur transiently and locally in specific tissues, such as the airway mucosa (94). By contrast, exhaled VOCs are integrated outputs of both systemic and airway-specific metabolism, capturing spatiotemporal metabolic dynamics across multiple organs (43).

Precision medicine has catalyzed innovations in molecular pathology, including advancements in the analysis of exhaled VOCs as a non-invasive diagnostic modality. VOCs in exhaled breath can provide rich chemical and metabolomic insights (95). For example, Chu et al. employed solid-phase microextraction–GC–MS with non-targeted analysis to identify signature VOCs in lung cancer cell lines (A549, PC-9, NCI-H460) and a normal lung epithelial line (BEAS-2B), both Results revealed three common discriminatory VOCs—ethyl propionate, acetoin, and 3-decen-5-one—present in all three lung cancer lines under resting conditions, but absent in normal cells. Under basal conditions and after glycolytic inhibition. Results revealed three common discriminatory VOCs—ethyl propionate, acetoin, and 3-decen-5-one—present in all three lung cancer lines under resting conditions, but absent in normal cells. Upon glycolytic inhibition, acetoin levels increased by 2.60–3.29 fold in all cancer cell lines, while remaining stable in normal cells. These findings suggest that glycolytic inhibition amplifies acetoin differentials between cancerous and normal cells, indicating its potential as a glycolysis-regulated biomarker for lung cancer detection (96). Chu et al. further elucidated the biosynthetic pathway of acetoin, demonstrating that glycolytic inhibition induces compensatory upregulation of the glutamine degradation pathway (97), resulting in elevated pyruvate levels. As pyruvate serves as a precursor for acetoin synthesis, its elevation consequently promotes acetoin accumulation (98). This interdisciplinary strategy—integrating molecular pathology, chemistry, and metabolomics via interventional VOC synthesis—offers a novel framework for lung cancer identification and may facilitate the development of new cytological diagnostic methods. Future studies will aim to co-culture normal and cancerous lung cells and analyze exhaled VOCs from patients under glycolytically controlled conditions, with the goal of advancing clinical translation (96).

However, inconsistencies in interdisciplinary terminology pose a critical barrier to effective collaboration (99). For example, in interdisciplinary studies involving molecular pathology, chemistry, and metabolomics, divergent definitions of the term “biomarker” have introduced systemic challenges in three key areas: failures in data integration (100), fragmented mechanistic validation (101), and barriers to clinical translation (102). These conflicts—stemming from discipline-specific biomarker validation criteria—prevent interoperability between databases, disrupt causal inference frameworks, and hinder clinical translation. For instance, Tian et al. identified nine exhaled VOCs characteristic of COPD using μGC–MS but did not elucidate their underlying mechanisms (103). In molecular pathology, biomarkers are expected to have a mechanistic association with disease onset and progression (104). Therefore, although the study demonstrated diagnostic value from chemical and metabolomic perspectives, its lack of mechanistic insight limits its clinical translatability.

Therefore, early consensus on terminology is essential to translate the “terminal information” carried by metabolites into actionable biomarker-based clinical decision-making. This shift is key to advancing precision medicine from phenomenological description toward mechanism-driven diagnostic and therapeutic integration. The foundation for standardizing interdisciplinary terminology lies in building a dynamic consensus system that balances disciplinary specificity with cross-domain universality. First, establish foundational norms based on international standards (e.g., ISO), and define core interdisciplinary semantics through ontological analysis (105). Second, adopt a hierarchical composite naming system that preserves disciplinary prefixes (e.g., “chemical-,” “cognitive-”) while utilizing a shared root lexicon to ensure traceability and cross-disciplinary interoperability (99). Third, develop a semantic association model using knowledge graph technologies to dynamically map conceptual relationships across disciplines, supporting ambiguity resolution and contextual adaptation (106). Finally, implement a collaborative governance framework that combines iterative Delphi consensus processes by expert panels with NLP-based large-scale analysis of term usage. This approach ensures continuous refinement of both academic rigor and practical communicability, supported by an open, traceable terminology database capable of real-time updates and feedback loops (107). This strategy aims to create a terminology ecosystem characterized by structural flexibility, evolutionary adaptability, and high disambiguation efficiency—serving as a foundational infrastructure for interdisciplinary knowledge integration.

#### 4.2.2 Advances in research methods and techniques

GC–MS remains the gold standard for comprehensive profiling of VOCs in exhaled breath due to its high stability, excellent separation efficiency, selectivity, sensitivity, and reproducibility (108). However, its clinical applicability is limited by substantial drawbacks, including the need for complex sample pretreatment, poor portability, high power consumption, lack of real-time analytical capacity, and high operational costs (109). These limitations render GC–MS unsuitable for point-of-care (POC) or rapid screening applications (110, 111). Proton transfer reaction–mass spectrometry (PTR-MS), by contrast, offers real-time, *in vivo* detection of trace-level VOCs with minimal sample preparation (112). Its high accuracy and specificity make it a promising tool for dynamic monitoring in clinical settings such as the ICU (113). Nevertheless, its analytical range is restricted to low-molecular-weight VOCs, potentially missing diagnostically relevant macromolecular biomarkers (114). Extractive electrospray ionization mass spectrometry (EESI-MS) enables direct, matrix-tolerant analysis of complex biological samples without pretreatment (115), showing promise for rapid diagnosis, especially in environments requiring operational simplicity and high throughput, such as ICUs. Its resistance to matrix effects enhances signal reliability in heterogeneous respiratory matrices. However, challenges remain in achieving robust quantitative reproducibility, which limits its current clinical deployment.

To address the limitations inherent to single-modality systems, recent research has emphasized the development of hybrid platforms, such as GC-PTR-MS, that combine the comprehensive compound identification capacity of GC–MS with the real-time (109), high-sensitivity capabilities of PTR-MS (113). Such systems aim to leverage complementary strengths to improve diagnostic coverage and adaptability. Furthermore, advances in miniaturized MS devices have significantly enhanced portability and usability, enabling integration into wearable gas sensor platforms (111). These sensors can serve as preliminary VOC screening tools, with positive cases referred to high-resolution MS backends for molecular confirmation, thereby reducing false positives and improving triage efficiency. A comparative assessment reveals clear trade-offs across platforms in terms of detection depth, analytical speed, cost-effectiveness, and standardization readiness. GC–MS excels in chemical resolution but suffers from logistical inflexibility; PTR-MS provides rapid and accurate detection but lacks breadth in VOC range; EESI-MS balances portability and matrix resilience but requires further validation for quantification (115). As clinical translation accelerates, standardized performance metrics, cost–benefit evaluations, and disease-specific suitability studies across these modalities will be critical. Future progress will depend on the coordinated advancement of hybrid instrumentation, clinical validation frameworks, and regulatory standards to ensure scalable and reproducible deployment in respiratory diagnostics (108).

The E-nose mimics the mammalian olfactory system by using an array of sensors to detect VOCs and applying pattern recognition algorithms to differentiate complex odor profiles (63). Due to its sensitivity, rapid response, and portability, the e-nose has been widely applied in respiratory disease research (116). However, the e-nose can only recognize disease-related breath patterns and lacks the capability to identify the specific chemical constituents responsible for these patterns (62). To overcome these limitations, the e-nose is often combined with MS, which provides high-resolution analysis of gas composition and enhances chemical specificity (117).

Substantial progress has been made in elucidating the mechanisms and identifying biomarkers of respiratory diseases through cellular and animal models. For example, dynamic monitoring of exhaled VOCs such as hexanal and pentanal has been shown to reflect oxidative stress during lung injury. The lipopolysaccharide (LPS)-induced acute lung injury (ALI) mouse model is commonly employed to mimic pulmonary inflammation. These models provide a valuable platform for screening anti-inflammatory agents and elucidating inflammatory signaling pathways, such as MAPK/NF-κB activation (118). Additionally, lung cancer organoid models have demonstrated high fidelity in biomarker research. Patient-derived organoids (PDOs) replicate the molecular and pathological features of primary tumors, producing VOC profiles closely resembling those of clinical samples. When integrated with multi-omics analysis, these models can reveal metabolic reprogramming pathways in the tumor microenvironment, supporting personalized treatment planning and drug sensitivity testing (119). Furthermore, advances in single-cell sequencing have enabled precise localization of VOC-producing cell populations. By comparing the transcriptomes of alveolar type II cells and cuprocytes, researchers identified specific cell subpopulations—such as type II alveolar cells enriched in lipid metabolism genes—as major sources of VOC production. This approach not only resolves cellular heterogeneity but also pinpoints molecular targets for targeted therapies (120).

Beyond integrating metabolomics and metagenomics data to confirm the biological origins of VOCs, Artificial Intelligence (AI) models are employed to fuse multidimensional datasets, including VOC profiles, imaging, and clinical indicators. Analysis of exhaled VOC data should adhere to a closed-loop framework encompassing screening, modeling, and verification. AI algorithms prioritized for this purpose should demonstrate interpretability (e.g., SHAP) (121), robustness against interference (e.g., Lasso-RF fusion) (122), and clinical adaptability (e.g., LSTM for real-time monitoring) (123). Concurrently, coordinated efforts to develop standardized databases and miniaturized detection devices are essential. To accelerate clinical translation and enable the shift from VOC signal detection to precision intervention, future validation studies should prioritize diseases based on mortality rates, the feasibility of VOC detection technologies, and existing gaps in clinical diagnostics. This prioritization aims to reduce preventable mortality by optimizing resource allocation and maximizing public health benefits within constrained research investments. Furthermore, stratified diagnostic and therapeutic guidelines informed by multi-omics markers should be developed, alongside fostering interdisciplinary collaboration (57). Although novel biosensors have been applied to respiratory disease research, their full potential remains unrealized. Despite initial advances in biosensing platforms altering the phenomic landscape of respiratory diseases, ongoing development and clinical translation efforts are required to fully realize their impact on research and therapeutic innovation.

## 5 Limitation

### 5.1 Data coverage constraints

Our exclusive reliance on WoSCC and PubMed may have omitted relevant studies from specialized databases (e.g., Embase, Scopus). The English-language restriction potentially excluded impactful non-English publications. Future investigations could expand retrieval to Embase, Scopus and utilize AI-assisted translation tools with domain-expert validation to mitigate language bias.

### 5.2 Bibliometric methodological boundaries

While effectively mapping research landscapes, bibliometric approaches cannot assess study quality or methodological rigor. Citation metrics may be influenced by journal policies and self-citation practices. These limitations necessitate complementary evidence synthesis methods: Structured qualitative appraisal (e.g., using ROBINS-I for risk of bias assessment) can evaluate the methodological soundness of high-impact studies identified through bibliometric networks. Dose–response meta-analyses may quantify clinical effect sizes of VOCs flagged as research hotspots, reconciling heterogeneous findings across studies.

This integrated approach creates a translational bridge: Bibliometrics identifies candidate biomarkers and knowledge gaps, while systematic review/meta-analysis validates their clinical credibility and quantifies diagnostic accuracy [e.g., pooled sensitivity/specificity of breath signatures for COPD exacerbations (61)].

### 5.3 Multidisciplinary integration challenges

Terminological and methodological heterogeneity across chemistry, immunology, and respiratory medicine complicates knowledge integration. Future interdisciplinary teams should employ ontology alignment tools (e.g., OLS API) and consensus frameworks like Delphi methods to standardize conceptual mappings.

Despite these constraints, this study provides a foundational mapping of research frontiers. Translation into clinical practice requires prospective multicenter trials using standardized VOC collection protocols (e.g., ATS/ERS guidelines) to validate biomarker reproducibility.

## 6 Conclusion

Over the past two decades, this comprehensive bibliometric analysis has mapped emerging trends and research hotspots in MS-based respiratory testing, highlighting its transformative potential for clinical diagnostics and research. MS is increasingly recognized as a non-invasive and highly sensitive technique for monitoring VOCs in exhaled breath, underscoring its clinical and research significance. Key advances encompass disease-specific VOC profiling, mechanistic insights linking VOCs to metabolic reprogramming, oxidative stress, and microbiome interactions, as well as methodological improvements enhancing diagnostic precision and reproducibility. Ongoing technological innovations continue to improve real-time, high-resolution VOC detection capabilities in MS. Integration of AI and machine learning facilitates predictive modeling and precise biomarker identification, while enhancing data analytics. To elucidate disease pathophysiology and enable precision medicine, future efforts will prioritize multi-omics integration. Advances in single-cell sequencing, organoid modeling, and biosensor technologies hold promise for bridging precision medicine and biomarker discovery.

Despite limitations related to database scope and the multidisciplinary nature of VOC research, this study provides a foundational roadmap to address knowledge gaps, foster international collaboration, and accelerate the clinical adoption of respiratory diagnostics. MS-based breath testing represents a cornerstone of next-generation respiratory disease management, driven by advances in technology, biomarker validation, and AI-enhanced analytics. However, critical challenges remain, including the lack of standardized protocols, limited biomarker specificity, incomplete understanding of biological mechanisms, immature technologies, and insufficient large-scale clinical validation. Addressing these issues requires urgent establishment of interdisciplinary collaborative alliances. Only through coordinated efforts among researchers, clinicians, and policymakers can exhaled VOC biomarkers—such as acetone and isovaleraldehyde, detected via MS—be translated into universal clinical tools, reshaping precise diagnosis and treatment paradigms for respiratory diseases. Specifically, researchers must develop ISO-certified sampling and analysis protocols to ensure data comparability across centers; clinicians should establish large prospective cohorts (≥10,000 patients) to evaluate VOC dynamics in response to treatment and exposure; policymakers are tasked with facilitating insurance coverage and expedited regulatory approval for portable, sensitive MS devices (e.g., MEMS Micro GC) to accelerate clinical translation.

## Glossary

ADH: Alcohol dehydrogenase
ALI: Acute Lung Injury
AI: Artificial Intelligence
ARDS: Acute Respiratory Distress Syndrome
COPD: Chronic Obstructive Pulmonary Disease
COVID-19: Corona Virus Disease 2019
ECM: Extracellular Matrix
EESI-MS: Extractive Electrospray Ionization Mass Spectrometry
EI: Electron Ionization
EMR: Electronic Medical Records
ESI: Electrospray Ionization
FeNO: Fractional Exhaled Nitric Oxide
GC: Gas Chromatography
GC–MS: Gas Chromatography–Mass Spectrometry
GC-TOFMS: Gas Chromatography-Time-of-Flight Mass Spectrometry
GC × GC-HRTOFMS: High-Resolution Gas Chromatography-Time-of-Flight Mass Spectrometry
IL-6: Interleukin-6
IPF: Idiopathic Pulmonary Fibrosis
LC: Liquid Chromatography
LLR: Log-Likelihood Ratio
LPS: Lipopolysaccharide
LSI: Latent Semantic Indexing
MCP: Multiple Country Publications
MS: Mass Spectrometry
m/z: Mass-to-Charge Ratio
OPLS-DA: Orthogonal Partial Least Squares Discriminant Analysis
OSA: Obstructive Sleep Apnea
PAH: Pulmonary Arterial Hypertension
PDOs: Patient-Derived Organoids
POC: Point-of-Care
PTR-MS: Proton Transfer Reaction-Mass Spectrometry
PTR-TOF-MS: Proton Transfer Reaction Time-of-Flight Mass Spectrometry
ROS: Reactive Oxygen Species
SCP: Single Country Publications
SESI-MS: Secondary Electrospray Ionization Mass Spectrometry
TB: Tuberculosis
TNF-*α*: Tumor Necrosis Factor-alpha
VOCs: Volatile Organic Compounds
WoS: Web of Science
WoSCC: Web of Science Core Collection
